# Extracellular Vesicles From a Model of Melanoma Cancer‐Associated Fibroblasts Induce Changes in Brain Microvascular Cells Consistent With Pre‐Metastatic Niche Priming

**DOI:** 10.1002/jex2.70094

**Published:** 2025-10-30

**Authors:** Mikayla Shelton, Chinedu A. Anene, Jeremie Nsengimana, Mahmoud K. Eldahshoury, Jacob G. Gillet‐Woodley, Ben Keane, Wayne Roberts, Julia Newton‐Bishop, James R. Boyne

**Affiliations:** ^1^ Centre for Biomedical Science Research, School of Health Leeds Beckett University Leeds UK; ^2^ PeptiMatrix, Biodiscovery Institute University of Nottingham Nottingham UK; ^3^ Population Health Sciences Institute, Faculty of Medical Sciences Newcastle University Newcastle UK; ^4^ University of Leeds, School of Medicine Leeds UK

**Keywords:** cancer‐associated fibroblasts, extracellular vesicles, melanoma brain metastasis, pre‐metastatic niche remodelling, tumour microenvironment

## Abstract

Malignant melanoma has one of the lowest 5‐year survival rates of any cancer and is characterised by its high invasiveness and metastatic potential, with especially poor outcomes in patients who develop brain metastases. Crosstalk between melanoma cells and cells of the tumour microenvironment (TME), including cancer‐associated fibroblasts (CAFs), is a central driver of disease progression. While the role of melanoma‐derived small extracellular vesicles (sEVs) in reprogramming stromal cells has been well documented, the reciprocal effects of CAF‐derived sEVs remain less clear. Here, using an in vitro model of melanoma CAFs, we show that CAF sEVs alter melanoma cells and fibroblasts to promote oncogenic traits and remodel endothelial cells, including brain microvascular cells, in ways consistent with early pre‐metastatic niche (PMN) changes. Multi‐omics cargo profiling revealed significant differential expression of proteins and RNAs linked to extracellular matrix organisation, vascular remodelling, and patient outcomes, with functional validation identifying THBS1 as an EV cargo that restrains endothelial sprouting while potentially promoting barrier destabilisation. Together, these findings suggest that CAF‐derived sEVs contribute to local and distal PMN remodelling, highlight their potential as therapeutic targets, and identify EV cargoes with promise as circulating biomarkers in melanoma.

## 1 Introduction

Melanoma is the 5th most common cancer diagnosed in the UK, and incidence continues to rise year‐on‐year, with a projected increase of 9% in the UK between 2023 and 2025 (Cancer Research UK 2023). Amongst solid tumours, melanoma has one of the highest propensities to metastasise to the brain, with autopsy data indicating that around 75% of melanoma patients die with brain metastases (Sloan et al. 2009). Melanoma brain metastasis is predictive of a poor outcome despite advances in systemic therapy. This is reflected by brain metastases having the highest AJCC melanoma stage (Stage IV M1d). Patients with Stage IV melanoma and no brain metastases (Stage IV M1c or less) have an overall survival rate of 58% at 3 years with the most effective melanoma treatment, a combination immunotherapy regimen of ipilimumab and nivolumab (Wolchok et al. 2017). This compares to a 3‐year overall survival rate of only 36.6% in patients with symptomatic brain metastases receiving the same treatment (Tawbi et al. 2021). Not only do brain metastases predict the worst outcome for patients, but we also lack biomarkers predicting metastasis to this site and response to immunotherapy.

Recent work has shed some light on the characteristics of melanoma brain tumours (Biermann et al. 2022), but very little is known about the reasons for melanoma's spread to the brain. Cancer‐associated fibroblasts (CAFs) are key components of the melanoma tumour microenvironment (TME) and have been shown to drive disease progression, metastasis, and immunotherapy resistance (Jenkins et al. 2022; Liu et al. 2022; Mazurkiewicz et al. 2022; Peng et al. 2023; Tian et al. 2022; Zheng et al. 2022; Ziani et al. 2021). CAFs exert these effects via their mechanical properties (Cao et al. 2019), secretion of cytokines, chemokines and other effector molecules (Mao et al. 2021), and also through extracellular vesicles (EVs), a group of membranous particles that are released from almost all cell types and found in most biological fluids (Bazzan et al. 2021). Small EVs (sEVs) have been the focus of significant research given their capacity to induce molecular changes via direct interaction with extracellular receptors on the cell's surface or become internalised and release biomolecular cargo into the intercellular space (Kwok et al. 2021). A series of elegant studies have demonstrated sEV‐mediated regulation of the local TME in melanoma via modulation of melanoma metabolism (Clement et al. 2020), angiogenesis (Cheng et al. 2017) and in a seminal piece of work, pro‐oncogenic immunoregulation (Chen et al. 2018). A key aspect of sEV‐mediated cell‐cell communication is that crosstalk can occur over much larger distances than is possible via bilayer‐unprotected metabolites (Lattmann and Levesque 2022), facilitated by sEVs entering both the lymphatic and blood circulatory systems. Indeed, there is a growing body of evidence demonstrating that melanoma‐derived sEVs are important players in the establishment of the distant pre‐metastatic niche (PMN) (García‐Silva et al. 2021). Compared with cancer cell‐derived sEVs, far less is understood about how CAF‐derived sEVs influence the establishment of local and distant PMN in melanoma.

The aim of this study was to delineate the influence of CAF‐derived sEVs on PMN formation and understand the underlying non‐cell autonomous mechanisms of changes to cellular components of the melanoma TME in the brain. Specifically, we have established and validated an in vitro model of normal dermal fibroblast (NDF) re‐education towards a CAF‐like phenotype and the successful isolation and characterisation of sEVs released by these cells. Here, we show that our model CAF sEVs are internalised and reprogram melanoma cells towards a more migratory and invasive phenotype while also driving pro‐tumourigenic changes in other cell types found in the local TME. Moreover, we show that CAF sEVs promote remodelling of hCMEC/D3 brain endothelial cells, an early event associated with the establishment of the brain PMN. To understand the role of CAF sEV cargo in these phenotypic changes, we performed small RNA‐seq and quantitative proteomic profiling, revealing a signature of sEV miRNA and proteins that include reported drivers of endothelial‐to‐mesenchymal transition (EndMT). Taken together, our data show that reprogrammed CAFs secrete sEVs that promote metastasis, remodel brain endothelial cells, and carry a distinct biomolecular signature linked to endothelial‐mesenchymal transition.

## 2 Materials and Methods

### 2.1 Cell Culture

NDFs were prepared from skin specimens collected from the Huddersfield Royal Infirmary through routine abdominal surgical procedures with National Health Service (NHS) Research Ethics Committee (REC) approval (ref no: 15/EM/0265) and informed consent from patients with no history of skin malignancy and isolated as described (Peake et al. 2024). Human melanoma cell lines A2058 and A375 were purchased from European Collection of Authenticated Cell Cultures. Primary HUVECs were purchased from Thermofisher. The hCMEC/D3 cell line was a gift from Dr Paul Meakin (University of Leeds). All cell lines were routinely tested and certified mycoplasma free. Fibroblast and A2058 cells were maintained in High Glucose DMEM (Sigma Aldrich) and A375 cells in RPMI 1640 Medium with L‐Glutamine (BioWhittaker), supplemented with 10% FBS (Sigma Aldrich) and 100U/mL penicillin/streptomycin (Gibco). Endothelial cells were grown in Endothelial Cell Growth Medium with SupplementMix (Promocell). Cells were routinely cultured at 5% CO_2_ and 37°C in T75 flasks (primary cell lines never grown to more than passage 5). Cells were counted using the Corning Automated Cell Counter (Corning). For generation of model CAFs, NDFs were seeded in flasks in 10% DMEM and left to attach and grow for at least 24 h. Media was then changed to DMEM supplemented with 5 ng/mL of recombinant human TGF beta 1 (TGFβ1) protein (Abcam) for 48 h. For treatment of cells with sEVs, cells were grown (0.3–3 × 10^5^ cells) in 6 or 24‐well plates for 24 h in EV‐free media, then treated with 3 × 10^3^ sEVs per cell, isolated from either A2058, A375, NDF or CAFs and incubated for 24–72 h.

### 2.2 sEV Isolation

Cell lines were grown to ∼80% confluency, then grown in serum‐free media for 16 h. 40–80 mL media was spun at 300 × *g*, then 4000 × *g*, and 0.2 µm filtered, then concentrated to 0.5 mL using the Amicon Ultra‐15 10 kDa Centrifugal Filter Unit (Sigma Aldrich) according to the manufacturer's protocol. 0.5 mL media was then run on an SEC qEVoriginal (Izon) according to the manufacturer's protocol, and fractions containing sEVs (fractions 7–11) were collected.

### 2.3 Nanoparticle Tracking Analysis

NTA measurements were performed on a Malvern NanoSight NS300 (NanoSight, Amesbury) with a Blue488 laser type and sCMOS camera. NTA 3.2 Dev Build 3.2.16 software was used to capture and analyse the videos. All samples were diluted 1:20–100 in EV‐free PBS (Gibco), and three measurements of 90 s were carried out for each sample at 22°C with syringe speed 75.

### 2.4 CellMask Orange sEV Staining

5 mg/mL of CellMask Orange Plasma Membrane Stain was diluted 1:1000 in EV‐free PBS (Gibco). 1:1 (v/v) of working stain was added to sEVs and incubated at 37°C for 10 min. sEVs underwent additional centrifugation and PBS wash steps with Amicon Ultra‐0.5 100 kDa Centrifugal Filter Unit (Sigma Aldrich) as described (Perez‐Riverol et al. 2025) to remove unbound dye. sEVs were quantified via NTA, and an equal number of sEVs were added to cells and left to incubate for 24 h. An equal volume of PBS with or without CellMask Orange was used as controls, and PBS dye‐only controls were processed identically to EV samples (labelled and subjected to the same dye‐removal steps) to control for residual dye. Cells were then used for downstream analysis via flow cytometry or immunofluorescence microscopy.

### 2.5 Transwell Co‐Culture of Cell Lines

NDFs/CAFs were treated with 20 µM of GW4869 (Sigma) for 24 h prior to coculture, with sEV secretion depletion being confirmed via NTA. 5 × 10^4^ endothelial cells were seeded into the bottom of a 24‐well plate. 5 × 10^4^ NDF/CAFs were seeded into 0.4 µm transwell inserts (Sarstedt) and incubated in 24‐well plates for 24 h before lysate and RNA were isolated.

### 2.6 Angiogenesis Tube Formation Assay

Ninety‐six‐well plates were coated with 30 µL of Geltrex LDEV‐Free Reduced Growth Factor Basement Membrane Matrix (Gibco) and incubated at 37°C for 30 min. Endothelial cells were seeded at 1.5 × 10^4^ cells per well. 25 ng/mL VEGF‐A165 (ThermoFisher) or 4% DMSO (ThermoFisher) were used as positive and negative controls of angiogenesis, respectively. Cells were left for 6–18 h to form networks of branching structures, captured on the EVOS XL Core Cell Imaging System (10x objective). Images were quantitatively analysed using AngioTool.

### 2.7 Transwell Assay

Cells were serum starved for 24 h prior to the assay. 1 mL serum containing media was added to the lower chambers of a 24 well plate with 8 µm TC‐inserts (Sarstedt), and 500 µL cell suspension (1 × 10^4^ cells) was added into the insert. Prior to seeding, inserts were coated with 50 µL Geltrex LDEV‐Free Reduced Growth Factor Basement Membrane Matrix (Gibco) and incubated at 37°C for 1 h. Cells were incubated for 6–24 h at 37°C, then fixed with ice cold 70% ethanol for 30 min. Insert membranes were stained in 0.2% crystal violet (Fisher Science) for 10 min, then mounted onto slides. Five random high‐power images were taken (EVOS XL Core Cell Imaging System, 4x objective) and an average was calculated using ImageJ.

### 2.8 Collagen Contraction Assay

Fibroblasts were resuspended so 3 × 10^5^ cells were present in 1.2 mL media. 600 µL of 3 mg/mL rat tail collagen I (ThermoFisher) was added to cells, then 21 µL of 1 M NaOH. 500 µL was added per well of a 24‐well plate and allowed to solidify for 20 min at 37°C, then 500 µL of media was added to each well. Gels were dissociated carefully from well walls using a 10 µL pipette tip, and cells were left to contract for 24 h. Images were quantified on ImageJ, normalising area of gel outline to the area of the entire well.

### 2.9 Mass Spectrometry

Protein from 500 µL pooled sEVs from multiple NDF donors and their TGFβ1‐transformed CAFs in duplicate were extracted using RIPA, as described in Supporting Information. Samples were analysed by Dr Kate Heesom (University of Bristol) using tandem mass tagging coupled with liquid chromatography mass spectrometry (TMT LC‐MS). The TMT‐labelled proteomic dataset was processed to ensure the accuracy and reliability of protein identifications. Protein codes were cross‐referenced with the UniProt database (www.uniprot.org) to verify their presence in the reviewed (Swiss‐Prot) dataset, ensuring high‐confidence annotations. Only proteins with valid UniProt accessions were retained for downstream analysis. Post‐acquisition, TMT reporter ion intensities were normalised using internal reference scaling and median normalisation across all channels to correct for loading and technical variation, enabling accurate quantification of relative protein abundance across samples. Differential protein expression was assessed using moderated *t*‐statistics with FDR correction. The EXOCARTA dataset was downloaded from exocarta.org. Gene identifiers from Exocarta were converted to UniProt IDs using the UniProt ID mapping tool to standardise protein identifiers and facilitate comparison with the experimental dataset. The processed TMT proteomic dataset was compared against the EXOCARTA list to identify overlapping proteins. Gene ontology (GO) enrichment analysis was performed using the PANTHER classification system (version 19.0, Gene Ontology Consortium). Enrichment analysis was conducted against the Gene Ontology Biological Process (GO‐BP) database using Fisher's exact test with false discovery rate (FDR) correction to account for multiple testing. GO terms with an FDR‐adjusted *p* value < 0.05 were considered significantly enriched. Search Tool for the Retrieval of Interacting Genes/Proteins (STRING) network analysis was used to generate predicted protein interactions. The mass spectrometry proteomics data have been deposited to the ProteomeXchange Consortium via the PRIDE partner repository (Perez‐Riverol et al. 2025) with the dataset identifier PXD063635.

### 2.10 RNA Sequencing

NDF/CAF sEVs were pooled from six donors in duplicate. 200 ng of small RNA sample was sent to Novogene for SE50bp sequencing (raw data is available via GEO). Raw reads were subjected to additional adaptor trimming, and low‐quality reads were removed using Trimmomatic v0.39 with parameters (WindowSize = 4, Required Quality > 20). Trimmed reads were aligned to the GRCh38/hg38 assembly of the human genome using Bowtie v1.3.1 with parameters (‐k 10 ‐v 0). HtSeq‐count v0.11.1 was used to quantify the miRNA abundance based on the human miRNA annotation (miRBase Release 22.1) with parameters (‐s no ‐a 10 ‐m union ‐–nonunique none). The expression levels of miRNA and mRNA were normalised by counts per million (CPM). Differential expression analyses between different groups were performed using the Limma–Voom method based on the Limma R package. For sEV samples, the differential expression was defined at *p* ≤ 0.01 due to the small number of detected RNAs across the samples.

### 2.11 Statistical Analysis

Data are expressed as the mean ± standard error of the mean from at least two independent experiments carried out in triplicate. Statistical analysis was performed using GraphPad Prism 8.4.2. Principal component analysis and volcano plots were created using R 4.2.2. An unpaired *t*‐test or one‐sample Wilcoxon signed‐rank test was used to compare the means of two independent groups. A one‐way ANOVA or Kruskal–Wallis ANOVA was used to determine statistical significance between the means of three or more independent experimental groups, followed by a Tukey's post‐hoc test or unpaired *t*‐test with Welch's correction. Significant results are denoted as: *p ≤ 0.05, **p ≤ 0.01, ***p ≤ 0.001, ****p ≤ 0.0001, or ns (not significant) = *p* > 0.05. Functional CAF‐derived EV effects are compared to both untreated control and as a comparison between NF‐ and CAF‐derived EVs.

## 3 Results

### 3.1 Establishment of a Melanoma CAF Model

CAFs are defined as persistently activated fibroblasts in the tumour stroma that influence many parameters of melanoma progression (Papaccio et al. 2021). When NDFs transition into CAFs, they take on characteristics similar to smooth muscle, becoming more contractile and migratory and increasing secretion of various ECM remodelling proteins (Maley et al. 2017). Whilst CAF populations between individuals and within the primary TME are heterogeneous, expression of certain markers has been identified to signify CAF activation from NDFs (Zhao et al. 2023). CAF activation is a complex process that can occur via multiple routes and through physical and biochemical stimulatory factors; however, transforming growth factor β (TGFβ) is widely reported to act as a potent inducer of CAF differentiation (Isella et al. 2015; Kalluri and Zeisberg 2006; Shi et al. 2020; Zhou et al. 2020). To mitigate heterogeneity between patient samples, and considering that CAFs are generally activated due to tumour‐derived signalling rather than intrinsic mutations, we sought to generate an in vitro model that exhibited CAF‐associated phenotypic properties and displayed characteristic markers.

**FIGURE 1:**
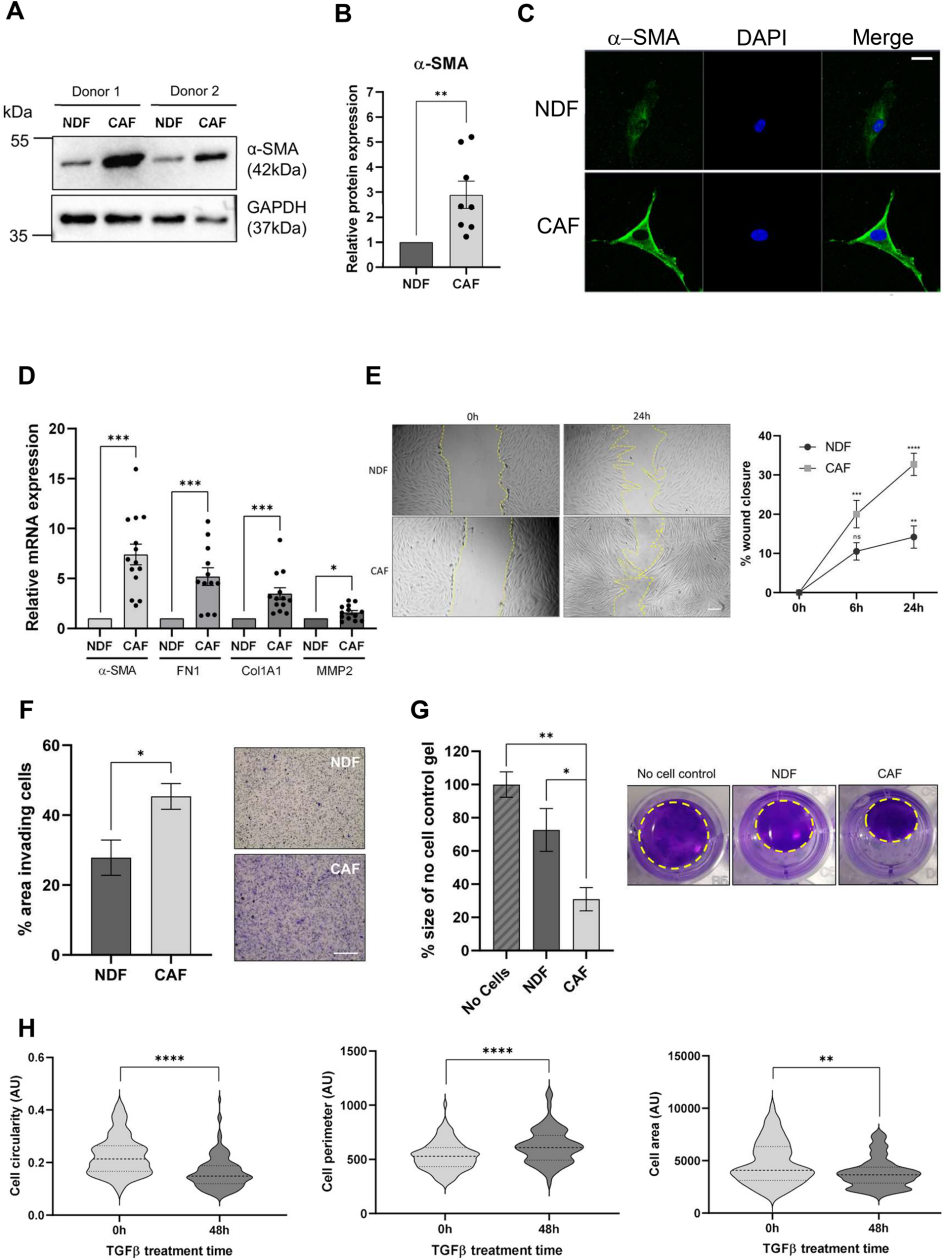
Characterisation of model CAFs treated with TGFβ1. (A) Representative western blot of α‐SMA expression in two donor populations treated with or without 5 ng/mL TGFβ1 for 48 h. (B) Quantification of α‐SMA protein expression normalised against GAPDH. (C) Representative immunofluorescence microscopy images of NDF or model CAFs stained with α‐SMA‐FITC and DAPI. Scale bar = 20 µm. (D) Relative transcript expression of characteristic CAF markers normalised against reference genes (GAPDH, TBP and RER1) in NDFs compared with model CAFs. (E) Representative images of a scratch wound assay, % wound closure in NDFs and model CAFs. (F) Quantified cell invasion as % area of transwell containing invading cells per field of view and representative images of transwell invasion assay for NDFs and model CAFs. Scale bar = 500 µm. (G) Quantification of collagen contraction over 24 h, relative to no cell control and representative images of collagen gel contraction of NDFs or model CAFs. Yellow dashed line = outline of gel. (H) Cell circularity, cell perimeter and cell area quantified in ImageJ at 0 and 48 h post TGFβ1 treatment. Quantitative morphometric analysis of >100 cells per condition confirmed statistically significant differences in area and circularity, consistent with a myofibroblast‐like phenotype. Dashed lines show median and quartiles.

To this end, primary NDFs were isolated from skin and cultured with TGFβ1 to re‐educate cells to a CAF‐like state. Consistent with previous observations (Melling et al. 2018; Casey et al. 2008), TGFβ1 induced an increase in protein expression of α‐SMA in treated NDFs, as evidenced by Western blot, IF and FACs analysis (Figure 1A,C and Figure S1D). α‐SMA upregulation persisted over at least two passages and following 96 h culture post‐TGFβ1 exposure, indicating stability of the induced phenotype (Figure S1H). To determine if our model CAFs displayed reported transcriptional changes, we analysed the expression of four TGFβ1‐regulated genes reported to be enriched in melanoma CAFs, namely FN1, COL1A1, MMP2 and ACTA2 (α‐SMA) (Melling et al. 2018; Liu et al). As can be seen in Figure 1D, we observed a significant increase in the transcript levels for each of these genes in our TGFβ1‐treated model CAFs compared with untreated controls. In addition, we also assessed the expression of several miRNAs previously reported to be differentially expressed in melanoma CAFs (Shelton et al. 2021; Melnik 2015). In each case, we observed a significant difference in the abundance of these transcripts in our model CAF cells compared with control (Figure S1E).

Next, we investigated changes in several phenotypic parameters consistent with reported *in* *vivo* characteristics of CAFs, including increased cell migration, invasion and crucially contractility within a 3D matrix (Sahai et al. 2020). As can be seen in Figure 1E,G, we observed a significant shift towards CAF‐like properties. Moreover, model CAFs had significant increases in ATP production and mitochondrial dehydrogenase activity, despite no changes to cell viability, suggesting increased proliferation (Figure S1A–C). Finally, morphological analysis of model CAFs reflected myofibroblast‐like changes, with longer protrusions giving decreased circularity and increased perimeter (Figure 1H). Together, these data demonstrate that TGFβ1‐treatment of primary NDFs generates CAF‐like cells that display biochemical and phenotypic parameters consistent with melanoma CAFs.

### 3.2 Model CAFs Release sEVs That Are Internalised by Cells Typical of the Local and Distal Melanoma TME

**FIGURE 2:**
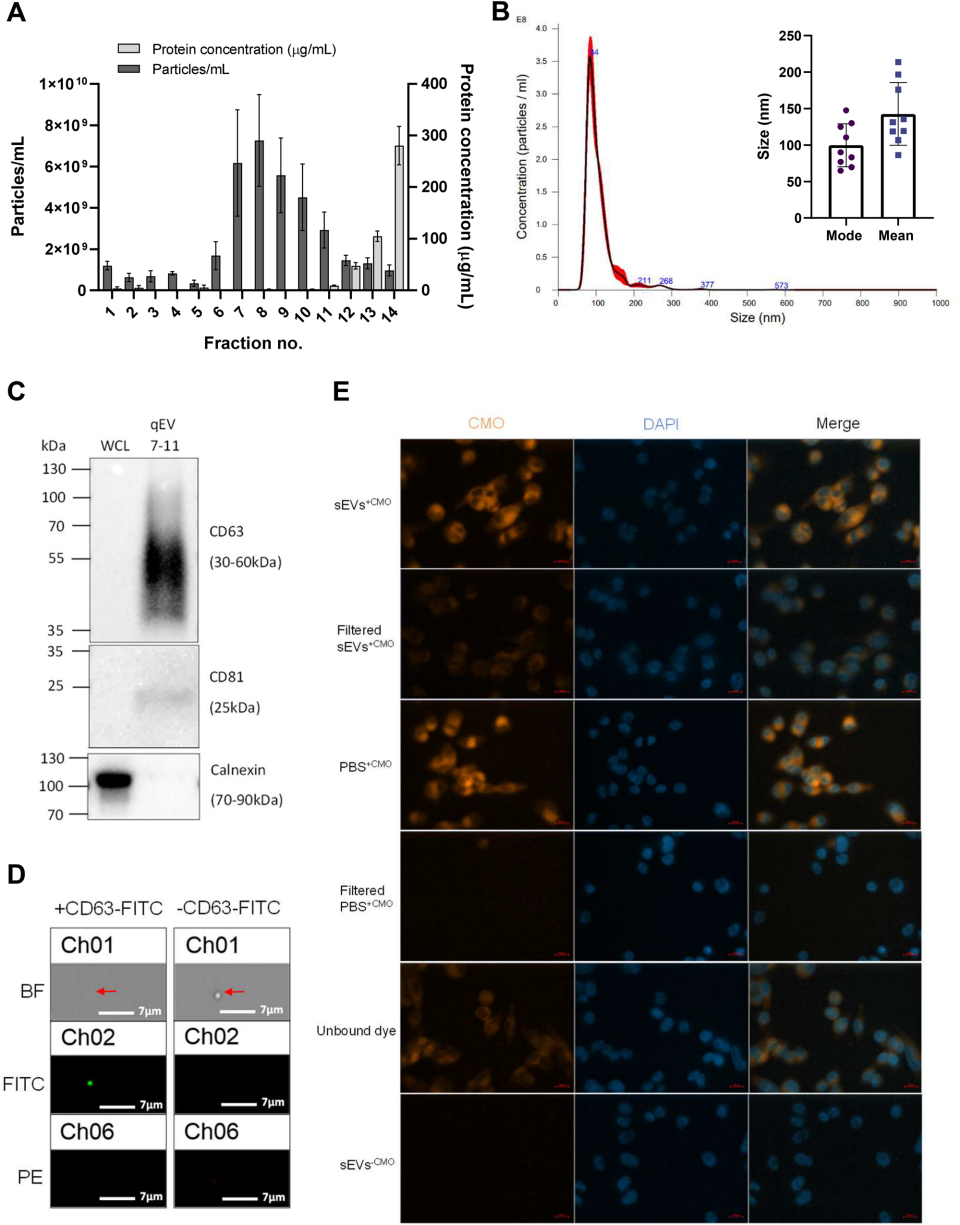
Characterisation of CAF sEVs isolated via SEC and their uptake into recipient cells. (A) Average particle and protein concentration of fractions 1–14 eluted from SEC of model CAF cell cultured media measured via NTA and micro‐BCA, respectively. Three 90‐s videos were captured per sample replicate for NTA. (B) A representative NTA size and concentration profile of combined fractions 7–11 from model CAFs isolated via SEC. Red error bars indicate SEM. Particle analysis revealed a mode size of 100 ± 27.9 nm and a mean size of 142 ± 40 nm (*n* = 3). (C) Western blot of model CAF SEC combined fractions 7–11 alongside model CAF whole cell lysate (WCL) against sEV markers. (D) Representative FlowSight images of sEVs stained with or without CD63‐FITC after gating for EVs based on area and aspect ratio. Scale bars = 7 µm. (E) Representative IF microscopy images of A375 cells co‐cultured with model CAF sEVs or EV‐free PBS stained with or without CMO, with or without unbound dye removed. PBS 'dye‐only' controls were processed identically to EV samples (labelled and subjected to the same dye‐removal steps) to control for residual dye. Scale bar = 20 µm.

We next sought to isolate and characterise sEVs released by our model CAF cells, observing MISEV guidelines (Welsh et al. 2024). To this end, TGFβ‐induced model CAF cells were cultured in EV‐free media prior to isolation of sEVs via size‐exclusion chromatography (SEC), which facilitates a high yield of sEVs, low variability between isolations, and a homogenous population with retained functionality (Sidhom et al. 2020). As seen in Figure 2A, particles were enriched in SEC fractions 7–11, and downstream analysis using NTA and TEM showed that particles from these collated fractions displayed an average size that fell within the 30–200 nm range characteristic of sEVs. NTA further showed a predominant particle population with a mode size of 100 ± 28 nm and a mean size of 142 ± 40 nm, consistent with the expected size distribution of small EVs (Figure 2B and Figure S2A,B). To confirm that isolated particles harboured reported sEV marker proteins, CD63 and CD81, western blot analysis was performed. As can be seen in Figure 2C, particles from SEC fractions 7–11 were enriched in sEV markers, while in contrast, the negative sEV marker, Calnexin, was absent, demonstrating that isolated particles were not contaminated with membranous structures derived from the intracellular endoplasmic reticulum. We next sought to confirm the presence of the canonical sEV marker protein, CD63, at the single particle level via imaging flow cytometry and in line with MIFlowCyt‐EV guidelines (Welsh et al. 2020). As can be seen in Figure 2D, we observed a robust signal for CD63 on single particles, and when plotting the intensity of gated particles, there is a clear population of CD63^+^ particles within the total population (Figure S2C,D).

In order to investigate if model CAF‐derived sEVs are internalised when co‐incubated with target cells, sEVs were stained with amphipathic CellMask Orange (CMO) dye (Takov et al. 2017), and CMO binding to sEVs was confirmed via amplified fluorescence polarisation compared with the unbound dye (Figure S2E). Stained sEVs were then co‐cultured with a range of cell lines that are representative of the melanoma TME, including the melanoma cell lines A375 and A2058, NDFs, HUVECs (as a model of local TME endothelial cells) and hCMEC/D3 (as a model of brain endothelial cells). In each case, fluorescence microscopy imaging showed diffuse CMO staining within cells co‐incubated with labelled sEVs, with a higher fluorescence signal in filtered sEVs compared to filtered PBS (Figure 2E and Figure S2F), data that was also confirmed and quantified via FACS analysis (Figure S2G–L). Together, these data demonstrate that model CAFs release sEVs that are internalised by a range of cells commonly associated with the melanoma TME.

### 3.3 Model CAF sEVs Drive Re‐Education of Melanoma TME Cells in vitro

**FIGURE 3:**
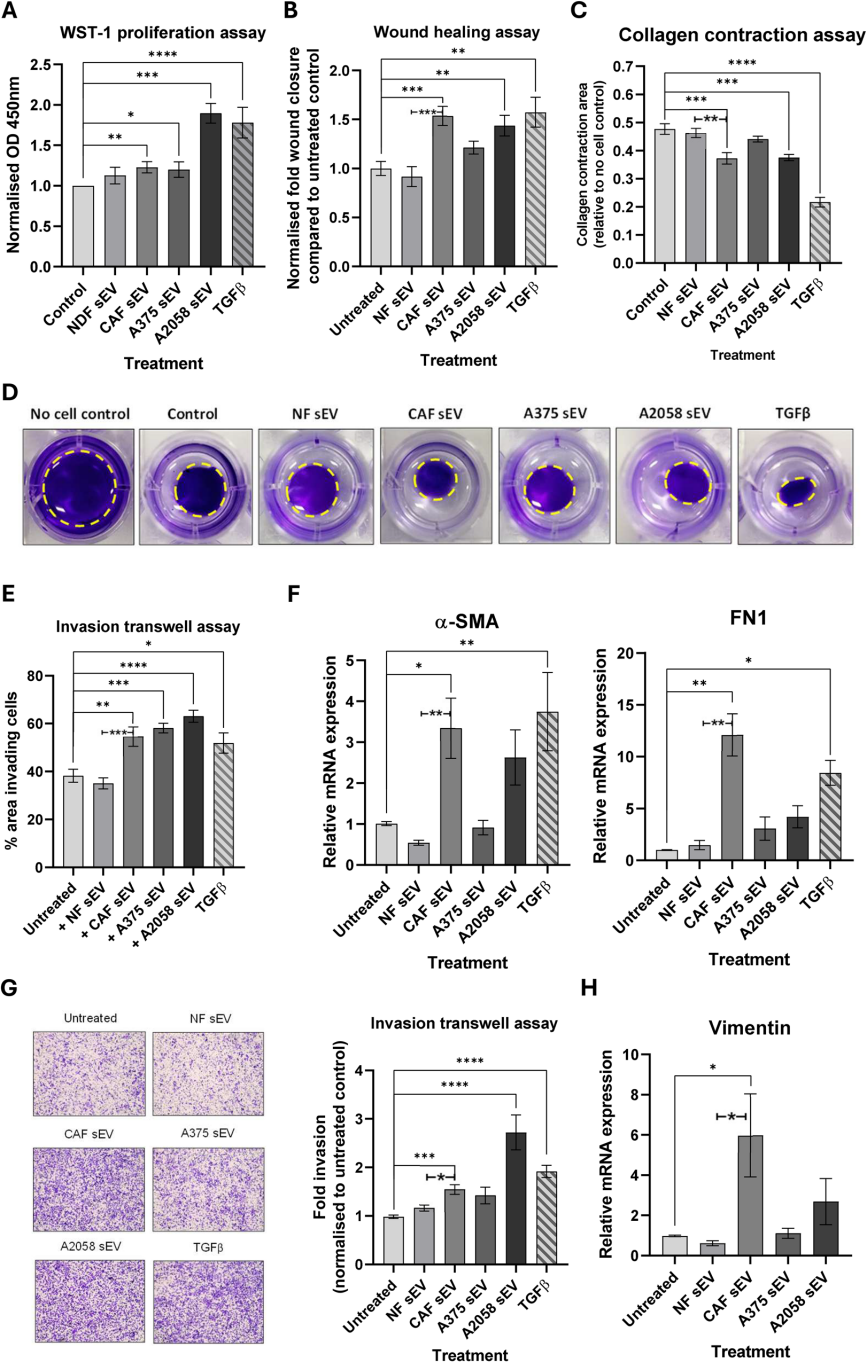
CAF sEV treatment affects multiple phenotypic parameters in dermal fibroblasts and melanoma cell line A375. (A) NDF co‐cultured with sEV were analysed via WST‐1 assay, and normalised fibroblast proliferation rate determined as optical density at 450 nm using 630 nm as a reference to measure formazan dye production. (B) Quantified sEV‐treated NDF migration compared with untreated control after 24 h. (C) Collagen matrix contraction after 24 h co‐culture of NDFs with sEVs relative to no cell control. (D) Representative images of NDF collagen contraction assay 24 h post indicated treatment (yellow dashed line = outline of gel). (E) Quantified cell invasion as fold invasion through transwell per field of view, normalised to untreated NDF control. (F) Relative transcript levels of CAF markers α‐SMA and FN1 in sEV‐treated NDFs. (G) Representative 24 h images of A375 transwell invasion assay alongside quantified cell invasion as fold invasion through transwell per field of view, normalised to untreated A375 control. (H) Relative expression of vimentin in sEV‐treated A375 cells.

After confirming that melanoma and other local TME cells internalise model CAF‐derived sEVs, we investigated how this uptake affects recipient cell properties compared to the uptake of sEVs from NDFs. In addition, as previous studies have shown that melanoma‐derived sEVs play a role in re‐educating cells within the TME (Kluszczynska and Czyz 2023), we also decided to isolate sEVs from the A375 and A2058 melanoma cell lines, which exhibit low and high invasive potential, respectively (Cheng et al. 2017; Giles et al. 2013), and determine how these might impact the NDF‐CAF axis. Initially, sEVs derived from each of these cell backgrounds were added to NDFs to determine how they impacted fibroblast activation. As can be seen in Figure 3A,E, sEVs from CAFs and the invasive melanoma line A2058 significantly increased proliferation, migration and contraction of NDFs when compared with sEVs isolated from NDFs and A375 cells. Moreover, CAF‐derived sEVs caused significant increases in transcript levels of α‐SMA and FN1 (Figure 3F) but did not significantly increase levels of MMP2 or Col1A1 (Figure S3C). Next, we examined how co‐culture with sEVs impacts the less invasive A375 melanoma cell line. Interestingly, we observed that model CAF‐derived sEVs caused a significant increase in the invasion potential of A375 cells (Figure 3G), but not migration in the absence of basement membrane (Figure S3D). Analysis of EMT transcript levels revealed increased levels of vimentin transcripts in cells co‐incubated with CAF‐derived sEVs (Figure 3H); however, there were no significant changes to SNAIL or TWIST (Figure S3E). These responses are therefore described as indicative of a partial EMT‐like shift. Taken together, these studies suggest that CAF‐derived sEVs can alter the metastatic properties of melanoma cells and re‐educate NDFs towards a CAF‐like phenotype, indicating a potential multi‐directional crosstalk facilitated by sEVs released from both melanoma and CAF populations within the TME.

### 3.4 Model CAF sEVs Drive an Angiogenic Switch in Endothelial Cells

Given our in vitro data demonstrating sEV multi‐directional crosstalk between melanoma cells and model CAFs, we next sought to elucidate whether melanoma and CAF sEVs might also impact a model of local TME endothelia. To this end, HUVEC cells were cultured with sEVs, and a tube formation assay was performed to assess angiogenic stimulation. As can be seen in Figure 4A,C, we observed a significant increase in branching index (indicative of a potent pro‐angiogenic stimulus) when HUVECs were co‐incubated with either model CAF or A2058‐derived sEVs that was absent for NDF‐derived sEVs. Next, we assessed the impact of CAF sEV co‐incubation on the abundance of pro‐angiogenic proteins in HUVECs (Figure 4D,F). We observed increased levels of numerous pro‐angiogenic proteins, including VEGF, PXDN, ANGPTL4 and TIMP‐1, a factor upregulated in colorectal cancer EVs that induces ECM remodelling in recipient fibroblasts (Kalluri and Zeisberg 2006). In the context of cancer progression, endothelial‐to‐mesenchymal transition (EndMT) is a key process in endothelial remodelling that compromises vascular integrity and promotes mesenchymal traits, enabling increased permeability and matrix remodelling (Fang et al. 2021). In parallel, qRT‐PCR of EndMT markers revealed a significant increase in ACTA2 (α‐SMA), while VIM (vimentin), TWIST and CDH5 (VE‐cadherin) did not reach significance (Figure 4G,K). Together, these results demonstrate that model CAF sEVs induce angiogenic potential and expression of pro‐angiogenic and EndMT markers in HUVECs.

FIGURE 4Model CAF sEV treatment changes angiogenic properties of HUVECs. (A) Representative angiogenesis branching assay images for HUVECs with treatment of sEVs or VEGF. Images were captured on the EVOS XL Core Cell Imaging System, at 10x objective and analysed using AngioTool (white line = explant area, yellow line = boundary of vessel, red line = vessel, blue dot = branching point). Quantified (B) branching index (no. of branching junctions per image) and (C) Vessel density (% area in explant area occupied by vessels). Statistical significance was determined for all using a one‐way ANOVA or a Kruskal–Wallis ANOVA, respectively. (D) Representative proteome profiler array membrane images for HUVECs with or without treatment of model CAF sEVs. Red boxes indicate reference spots in duplicate, green boxes indicate negative control spots. (E) Heat map of angiogenesis‐related protein abundance quantified via integrated density of model CAF sEV‐treated HUVECs, normalised to untreated. (F) Protein abundance of angiogenesis‐related proteins of model CAF sEV‐treated HUVECs, normalised to untreated HUVEC values. Statistical significance was calculated using a one‐sample Wilcoxon signed‐rank test. Quantification of (G) α‐SMA, (H) vimentin, (I) SNAIL, (J) VE‐Cadherin and (K) CD31 transcript expression via qPCR in HUVEC cells. Significance was quantified with a one‐way ANOVA. Range bars indicate SEM (*p ≤ 0.05, **p ≤ 0.01, ***p ≤ 0.001, ****p ≤ 0.0001).

**Figure:**
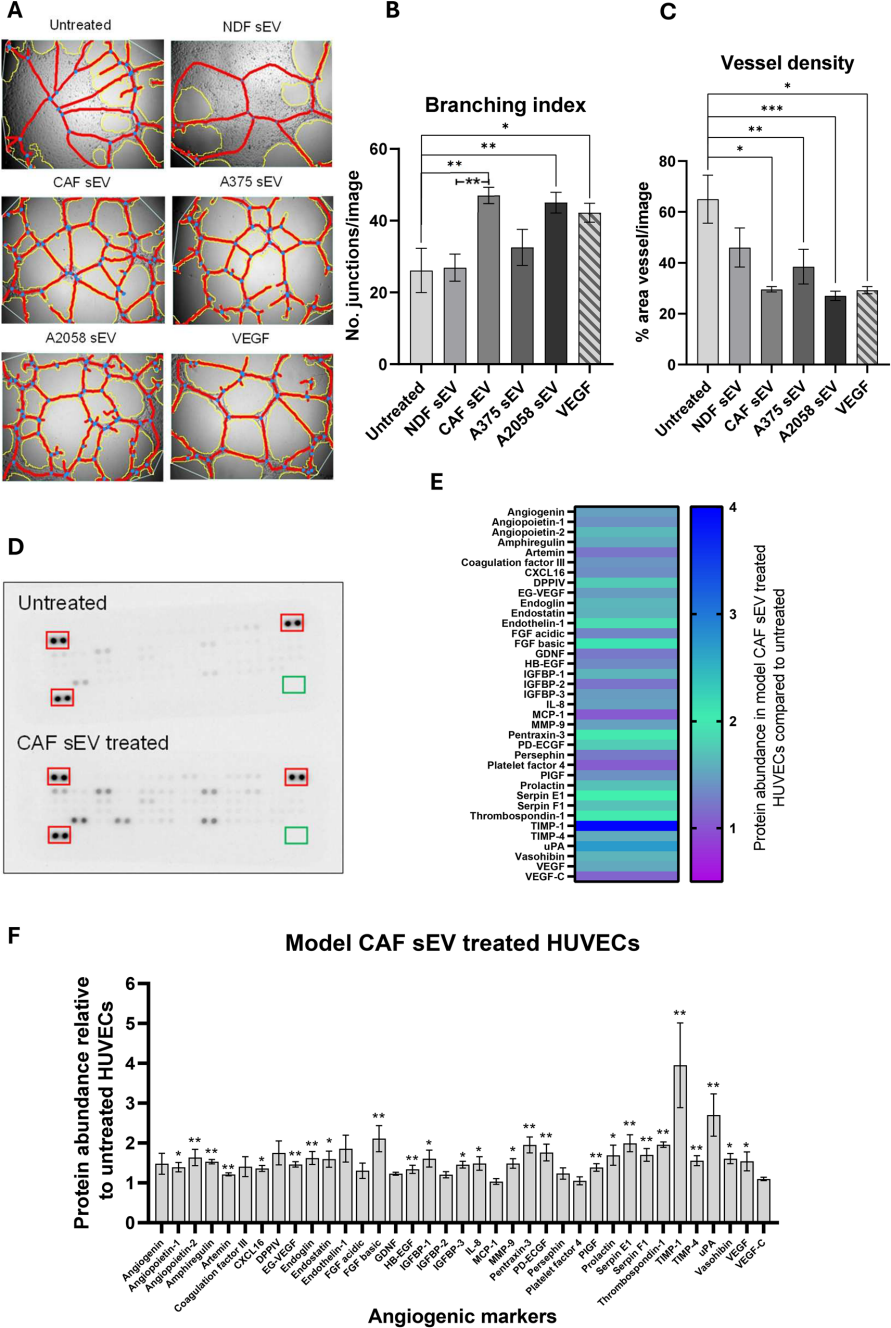

**Figure:**
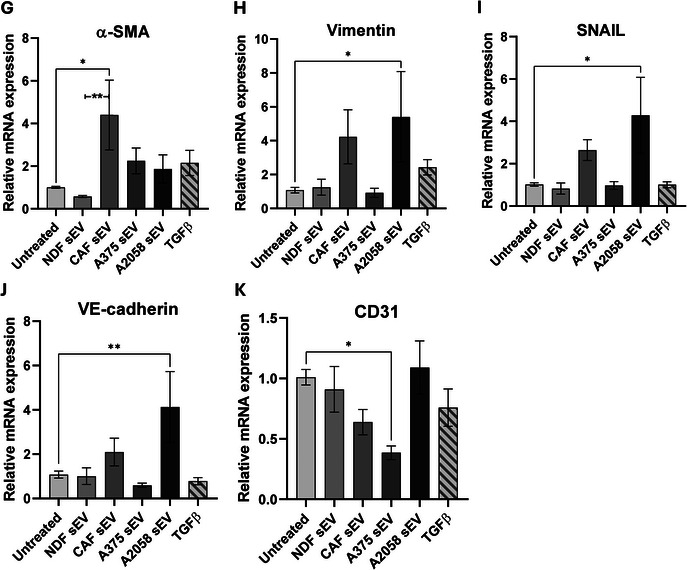

### 3.5 Model CAF sEVs Drive Re‐Education in a Model of the Brain PMN

Melanoma exhibits tropism towards the brain, with around half of all deaths from the disease presenting brain metastases (Abate‐Daga et al. 2018). Various phenotypic and transcriptional events have been previously associated with melanoma brain metastasis, including destabilisation of tight junctions, EndMT and activation of TGFβ/PDGFβ/PDGFRβ signalling (Zhang et al. 2022; Hashemi et al. 2022). Having shown that co‐culture with model CAF sEVs impact HUVECs, we were keen to investigate how they impacted an in vitro model of early BBB disruption—a key event in establishment of the brain PMN—using the human brain microvascular endothelial cell line, hCMEC/D3.

**FIGURE 5:**
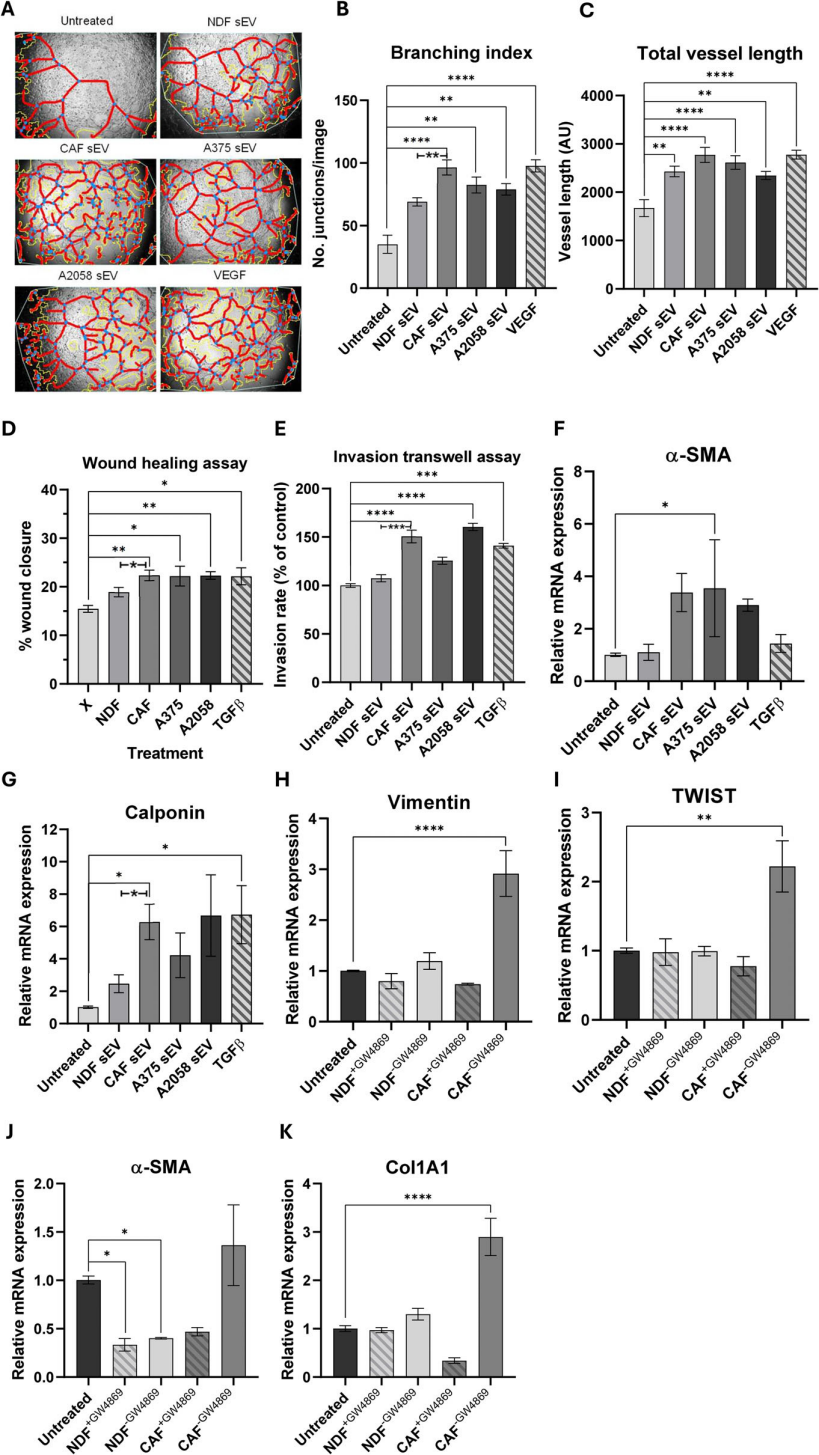
Model CAF sEVs drive re‐modelling of brain endothelial cells. (A) Representative angiogenesis branching assay images for hCMEC/D3s with treatment of sEVs. Images were captured on the EVOS XL Core Cell Imaging System, at 10x objective and analysed using AngioTool (white line = explant area, yellow line = boundary of vessel, red line = vessel, blue dot = branching point). Quantified (B) branching index (no. of branching junctions per image), (C) Total vessel length (sum of all vessel lengths in pixels). (D) Quantified cell migration as % wound closure in treated hCMEC/D3 cells. (E) Quantified cell invasion as % area invading cells, normalised to the untreated control. Quantification of (F) α‐SMA and (G) Calponin gene expression via qRT‐PCR in hCMEC/D3 cells. Quantification of (H) Vimentin, (I) TWIST, (J) α‐SMA and (K) Col1A1 gene expression via qRT‐PCR in hCMEC/D3 cells cocultured with NDF or model CAFs pre‐treated with or without GW4869.

Evaluation of pro‐angiogenic potential via the tube formation assay revealed that model CAF sEVs enhanced tube formation capacity in hCMEC/D3 cells, with significantly increased branching index and total vessel length per image (Figure 5A,C and Figure S5A–D). Furthermore, sEVs from both model CAFs and invasive melanoma line A2058 caused a significant increase in both the migratory and invasive properties of the endothelial cells, a phenomenon not observed with NDF sEV treatment (Figure 5D,E and Figure S5E,F). In addition, mesenchymal EndMT markers were evaluated via qRT‐PCR, which revealed increases in model CAF sEV‐treated samples compared to the untreated control (Figure 5F,G and Figure S5G–M).

To better understand how continuous exposure to sEVs released by NDFs and CAF model impacted hCMEC/D3 cells, fibroblasts and endothelial cells were co‐cultured in a transwell assay, with and without attenuation of sEV secretion. First, we confirmed that the neutral sphingomyelinase inhibitor, GW4869, could successfully attenuate sEV secretion in both NDFs and model CAFs (Figure S5N). We then indirectly co‐cultured fibroblasts with and without GW4869 pretreatment with hCMEC/D3 cells and analysed the expression of EndMT markers via qPCR. As can be seen in Figure 5H,K, we observed a significant increase in vimentin, TWIST and Col1A1 transcripts in hCMEC/D3 cells co‐incubated with model CAFs, which was absent in GW4869‐treated samples. Together, these data demonstrate that sEV release from model CAFs drives transcriptional and phenotypic changes in an *in* *vitro* model of the BBB.

### 3.6 Model CAF‐Derived Small Extracellular Vesicles Are Enriched in Pro‐Metastatic Cargo and Mirror Features of Melanoma Patient Plasma

Small extracellular vesicles (sEVs) mediate their functional effects on target cells through several complementary mechanisms, the most widely studies being direct delivery of bioactive cargo, such as mRNAs, non‐coding RNA (ncRNAs); in particular, miRNA, proteins and lipids, which can reprogram gene expression and modulate signalling pathways in recipient cells (Mathieu et al. 2019; Valadi et al. 2007). To investigate the biomolecular cargo of CAF‐derived sEVs and determine how it differs from that of NDF‐derived sEVs, we used a multi‐omics approach combining quantitative TMT‐based LC‐MS and small RNA sequencing to assess protein and miRNAs, respectively. TMT LC‐MS was performed on sEVs isolated from NDF and CAFs. Following filtering for common contaminants and low‐confidence hits, alignment was performed with the EXOCARTA proteome database (Keerthikumar et al. 2016), which confirmed significant enrichment of validated sEV proteins within our dataset (Figure 6A). Next, we compared normalised abundancies for each sample and identified 31 proteins that were significantly different between NDF and CAF‐derived sEVs. Thrombospondin‐1 (THBS1) exhibited the greatest enrichment in CAF‐derived sEVs. In addition, several other proteins were significantly altered, including increased PXDN (vascular peroxidase 1), biglycan (BGN), and decreased decorin (DCN) (Figure 6B). To explore the functional implications of proteins enriched in CAF‐derived sEVs, gene ontology (GO) analysis was performed on EXOCARTA‐validated proteins, which identified GOs consistent with CAF biology (Figure S6A). Next, we carried out GO analysis for proteins significantly increased in our model CAF‐derived sEVs; again, we observed GOs associated with CAFs but strikingly GOs associated with vascular regulation were also overrepresented (Figure S6B).

FIGURE 6CAF‐derived small extracellular vesicles are enriched in pro‐metastatic cargo and recapitulate features of melanoma patient plasma‐derived sEVs. (A) The TMT‐labelled proteomic dataset was filtered for high‐confidence UniProt‐reviewed proteins and compared with the EXOCARTA database, revealing that approximately 54% of the identified proteins were associated with extracellular vesicles. (B) Volcano plot of TMT LC‐MS analysis of protein abundance from model CAF sEVs compared to NDF sEVs. (C) THBS1 ELISA validation. Normal dermal fibroblasts (NDFs) were treated with TGF‐β1 (5 ng/mL, 48 h) in the presence of scrambled siRNA (Scramble) or THBS1‐targeting siRNA (THBS1kd). EVs were isolated from conditioned media, lysed, and THBS1 levels quantified by ELISA. *n* = 3 (left panels). EVs isolated from scramble‐ or THBS1‐siRNA‐transfected fibroblasts were applied to hCMEC/D3 endothelial cells seeded on Geltrex‐coated 96‐well plates. Tube formation was imaged using a Motic AE2000 microscope and analysed with WimTube (Wimasis) (centre panels). hCMEC/D3 cells treated with EVs from scramble‐ or THBS1‐siRNA‐transfected fibroblasts were subjected to scratch assays. Wound closure was monitored over 24 h, and migration quantified using ImageJ. *n* = 3 (D) Volcano plot of small RNA‐seq analysis of miRNA upregulated (red) or downregulated (blue) in model CAF sEVs compared to NDF sEVs (left panel). Z‐score hierarchical clustering heat map visualisation of differentially expressed miRNA in model CAF or NDF sEVs (right panel). (E) Volcano plot of non‐miRNA gene identified in our small RNA‐seq, analysed as described above. (F) Prognostic value of the top hits from *in* *vitro* studies were assessed against the LMC. Kaplan–Meier curves were plotted and Cox proportional hazards regression applied, setting high expression as reference. Melanoma‐specific survival was analysed ignoring the minority of deaths that were from non‐melanoma causes (*n* = 666). (G) Previously published data for miRNA‐seq analysis of melanoma patient blood plasma (GSE150956) were interrogated to determine transcript levels of miRNA enriched in model CAF‐derived sEVs.

**Figure:**
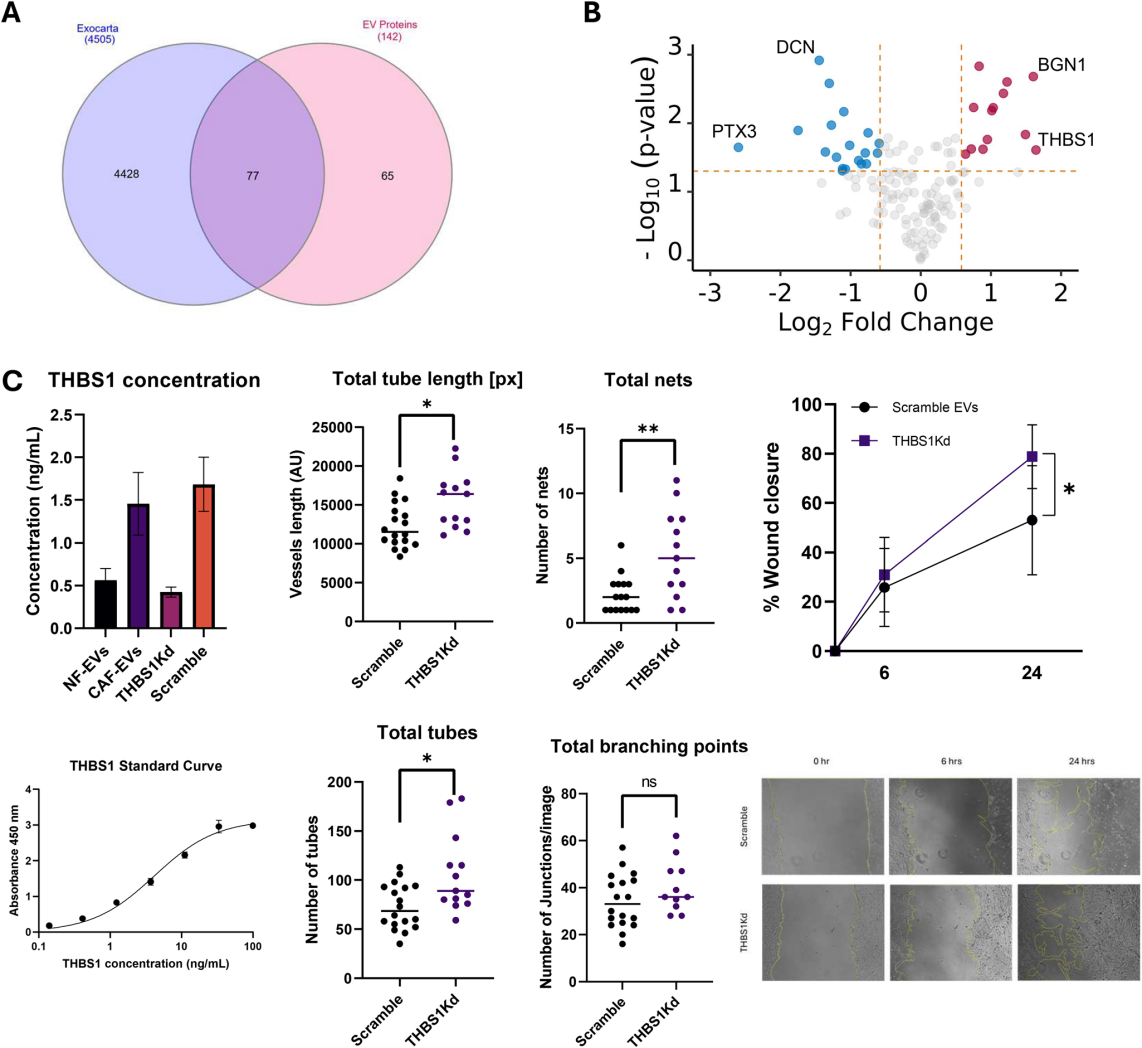

**Figure:**
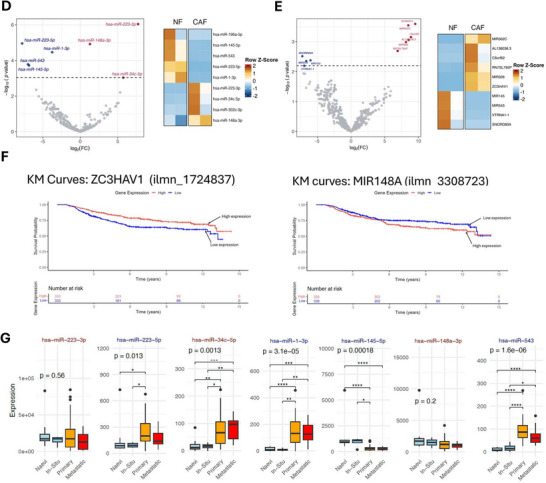

Given the enrichment in CAF‐derived sEVs and the well‐established but context‐dependent roles of THBS1 in angiogenesis, we chose to further validate and functionally investigate this protein. ELISA confirmed a significant increase in EV‐associated THBS1 following TGFβ1 education of fibroblasts, consistent with our proteomics data, and a significant reduction in THBS1 cargo in EVs from TGFβ1‐treated fibroblasts transfected with THBS1 siRNA compared with scramble control (Figure S6C). Functionally, EVs from THBS1‐depleted CAFs induced significantly greater endothelial tube length in angiogenesis assays compared with control EVs (Figure 6C and Figure S6C), and also promoted increased endothelial migration in scratch assays (Figure 6C). These findings suggest that THBS1 normally acts to restrain endothelial sprouting and motility. This is consistent with the reported dual and context‐dependent roles of THBS1 in vascular biology, where it can inhibit endothelial cell migration and sprouting via CD36/CD47 signalling, yet also activate latent TGFβ to promote junctional destabilisation.

Next, we performed small RNA sequencing on total RNA from NDF‐ and CAF‐derived sEVs to identify transcripts with significantly different expression levels between the two groups. As can be seen in Figure 6D, miR‐223‐3p, miR‐148a‐3p and miR‐34c‐5p were enriched and miR‐223‐5p, miR‐1‐3p, miR‐543 and miR‐145‐5p were decreased in CAF‐derived sEVs compared with NDF control, respectively. Interrogation of our small RNA‐seq data also revealed significant differences in other, non‐miRNA, genes (Figure 6E), including enrichment of ZC3HAV1. An emerging hypothesis is that sEV released by CAFs (and other tumour cells) may influence the PMN by entering the circulation and impacting on distal tissues (Costa‐Silva et al. 2015; Adler et al. 2019; Zhao et al. 2020). To this end, we were interested in determining if any of the protein or genes enriched in our model CAF‐derived sEVs were increased in the blood plasma of melanoma patients and thus might have clinical relevance. Melanoma datasets present in The Cancer Genome Atlas (TCGA) and The Human Protein Atlas were of limited utility as these mostly focus on tumour tissue, rather than patient plasma. Instead, we turned to a recent study that reported proteomic profiling of tumour and non‐tumour‐derived circulating sEVs in the plasma of melanoma patients with patient follow up for progression of disease (Pietrowska et al. 2021). Interestingly, this study identified thrombospondin‐1 as significantly enriched in melanoma tumour‐derived sEVs, which aligns with our quantitative proteomics analysis of our model CAF‐derived sEVs. More strikingly, of the 150‐proteins identified by the authors at melanoma‐specific thrombospondin‐1 was in the top 5 of a sub‐set of proteins that were predictive for metastatic progression of disease (Pietrowska et al. 2021). Next, to ascertain if CAF sEV‐enriched RNAs were reflected in clinical datasets, we returned to our previously published transcriptomic data from the primary melanomas of 703 patients, which comprises part of the Leeds Melanoma Cohort (LMC) (Muralidhar et al. 2019). Unfortunately, the HT12.4 array utilised in this study does not contain probes for all the ncRNAs identified in our RNA‐seq data, but we did observe a significant correlation between expression of ZC3HAV1 and miR‐148a and patient survival in primary melanoma tumour samples (Figure 6F). Interestingly, while increased levels of miR‐148a predicted reduced survival, ZC3HAV1 expression exhibited an inverse correlation. As RNA‐seq data from melanoma patient plasma is mostly unavailable from TCGA, we revisited a recent study that reported blood plasma miRNA expression data from individuals with invasive melanoma or related benign phenotypes (Love et al. 2024) and interrogated these data to investigate if any of the differentially enriched miRNAs identified in our CAF‐derived sEVs were significantly altered in melanoma patients with metastatic disease. As can be seen in Figure 5, 6/7 miRNAs identified in our RNA‐seq data were significantly altered in melanoma patient plasma, with miR‐145‐5p and miR‐34c‐5p displaying correlative enrichment in both datasets. Collectively, these data report model CAF‐derived sEV‐enriched protein and RNA, reveal biological functions for these related to PMN remodelling, and include proteins and genes associated with clinical melanoma studies, and patient outcomes.

## 4 Discussion

CAFs are increasingly recognised as a heterogeneous population whose functions are highly context‐dependent, shaped by tumour‐intrinsic signals, tissue type and local environmental cues (Sahai et al. 2020). In melanoma, where changes to the ECM, immune modulation, and vascular remodelling are fundamental to metastatic spread, CAFs are uniquely poised to support these transitions (Costa et al. 2018; Gieniec et al. 2019). Here we opted to investigate how CAF‐derived sEVs might contribute to modulation of the TME by establishing a simple, *in* *vitro* melanoma CAF‐like cell model derived from primary dermal fibroblasts and based on previously reported studies (Melling et al. 2018; Casey et al. 2008; Chen and Thibeault 2012). This approach enabled us to work with consistent cell populations, which displayed persistent α‐SMA induction over multiple passages and increased expression of CAF‐associated genes such as FN1, COL1A1 and MMP2, as well as successfully isolating and characterising sEVs for downstream analysis and functional assays (Figures 1 and 2). TGFβ1 was chosen as it is a well‐established inducer of the myoCAF phenotype (Öhlund et al. 2017); however, it represents only one axis of CAF heterogeneity. While our data demonstrate a convincing reprogramming of NDFs, we did not assess additional CAF markers such as FAP, TAGLN or POSTN in this study, and our model does not capture the full spectrum of CAF subtypes reported *in* *vivo*. Distinct CAF populations (e.g., myCAFs, iCAFs) are likely to release sEVs with differing cargo and functions, and fibroblasts activated by alternative cues such as IL‐1 or IL‐6 may produce vesicles with distinct phenotypes. Comparing such models will be an important avenue for future work.

Whereas early studies on sEV‐mediated tumourigenesis focused on unidirectional effects of cancer cell‐derived vesicles, contemporary models propose that cells within the TME ‘co‐evolve’ via multi‐directional crosstalk (Naito et al. 2022; Zhang et al. 2024; Han et al. 2021). Our *in* *vitro* cell line data support this model, specifically the observation that sEV released by our model CAFs not only promote significant increases in mesenchymal‐associated phenotypes in melanoma cells but also remodel endothelial cells and non‐activated NDFs (Figures 3 and 4). Importantly, CAF‐derived sEVs were not unique in this effect, as widely reported elsewhere, melanoma‐derived sEVs were also able to drive similar phenotypic changes (Peinado et al. 2012; Biagioni et al. 2021; Hu and Hu 2019; Felicetti et al. 2016; Matsumoto et al. 2017). In interpreting our results, it is important to consider the biological differences between the two melanoma cell lines used in this study. A2058 cells are widely characterised as more invasive and metastatic compared with A375 cells, which exhibit a less aggressive phenotype (Cheng et al. 2017; Giles et al. 2013). This is consistent with our observations that A2058‐derived sEVs induced stronger fibroblast activation and endothelial remodelling than A375‐derived sEVs. Moreover, A2058 cells display a more mesenchymal‐like phenotype with higher basal expression of EMT markers, whereas A375 cells retain more epithelial‐like characteristics. These intrinsic differences likely influence both the cargo composition of melanoma‐derived sEVs and the susceptibility of each line to reprogramming by CAF‐derived sEVs. For example, the increased invasive potential we observed in A375 cells following CAF sEV treatment may reflect a greater capacity for CAF‐derived signals to push an epithelial‐like line towards a more mesenchymal phenotype, whereas A2058 cells, already mesenchymal, may be less plastic in this respect. Together, these distinctions highlight that melanoma cell‐intrinsic state can modulate the extent and nature of sEV‐mediated crosstalk within the tumour microenvironment. This mutual reinforcement suggests a dynamic co‐evolution, potentially establishing a self‐perpetuating feedback loop. It highlights the TME as a Gordian knot of intercellular crosstalk, intricately involving cancer, stromal, endothelial and immune cell populations alike (de Visser and Joyce 2023). Indeed, longitudinal profiling of co‐cultures that incorporate macrophages (Quaranta et al. 2024) and other resident immune cells alongside the continued development of microfluidic organotypic models (Poddar et al. 2024; Deipenbrock et al. 2025) will be crucial for fully exploring and elucidating the biology underpinning TME co‐evolution and the contribution of sEVs to this process.

Studies investigating the impact of CAF‐derived sEVs on endothelial cells are somewhat limited but not without precedent (Miaomiao et al. 2023; Li et al. 2020). We observed a significant activation of angiogenesis in HUVEC cells cultured with CAF‐derived sEVs. This effect included increased expression of multiple angiogenic markers, including VEGF, once data were normalised to untreated HUVEC controls (Figure 4F). This effect was similar in magnitude to the responses elicited by A2058 sEVs and VEGF and led to a general increase in pro‐angiogenic markers (Figure 4F). We were particularly interested in how CAF‐derived sEVs might impact sites of distal PMN, particularly the brain, and so expanded our analysis to include the human brain microvascular cell line, hCMEC/D3, as a simple model of the blood‐brain barrier (Weksler et al. 2013). We observed similar activation of angiogenesis in hCMEC/D3 cells alongside increases in cell migration and transcriptional changes in the expression of genes associated with EndMT (Figure 5), with co‐culture assays inducing significant changes in Col1A1 compared with the non‐significant increase observed in HUVECs following the addition of purified CAF sEVs (Figure S3C). We interpret these transcriptional changes as preliminary evidence of EndMT, while acknowledging that protein‐level confirmation will be needed in future work. Seminal research demonstrating the tropism of cancer‐derived small extracellular vesicles (sEVs) for the lung, liver, and brain (Hoshino et al. 2015) laid the groundwork for understanding the contribution of circulating tumour‐derived sEVs to the modulation of the PMN. While this concept is now well‐established, most subsequent studies have focused on the impact of cancer‐derived sEVs (Li et al. 2024), including a recent article that describes a role for sEVs released by the brain‐seeking variant of triple‐negative breast cancer MDA‐MB‐231 cells in the brain PMN (Busatto et al. 2025). However, there are relatively few studies considering the role of CAF‐derived sEVs in distal PMN remodelling (Kong et al. 2019), despite the recognition now given to the importance of these cells in disease progression (Gieniec et al. 2019; Dong et al. 2023; Sahai et al. 2020; Kalluri 2016). Accordingly, we frame our findings as evidence of endothelial remodelling consistent with early PMN changes, rather than definitive proof of BBB disruption or PMN establishment. Further work using integrated BBB models that incorporate pericytes, astrocytes, and permeability assays will be required to extend these observations. It is also important to note that our experiments assessed Col1A1 induction in endothelial cells using two complementary approaches: in hCMEC/D3 cells, co‐culture with model CAFs in the presence or absence of GW4869 demonstrated that the effect was EV‐dependent (Figure 5K), whereas in HUVECs, EVs were first purified from CAF‐conditioned media and then applied directly (Figure S3C). The use of different endothelial models and experimental designs likely accounts for the observed differences in Col1A1 responses, while reinforcing that CAF‐derived EVs can modulate ECM‐related gene expression in both local and brain endothelial models.

To characterise the protein and miRNA content of CAF‐derived sEVs, we performed quantitative proteomics and small RNA sequencing. As expected, our proteomics data was enriched with widely reported sEV proteins, and both NDF and CAFs exhibited GOs associated with ECM organisation, wound healing and cell adhesion. Interestingly, when we investigated GOs based on those proteins that were significantly increased in our model CAFs, several ontologies relating to vascular remodelling reached significance (Figure 6C). However, we note that the number of significantly enriched proteins was modest and interpret these ontologies as exploratory. STRING analysis of CAF‐enriched proteins revealed a tightly interconnected core of ECM proteins along with modules anchored in vascular remodelling and haemostasis, suggesting that CAF‐enriched sEV proteins may fulfil specific functions. Indeed, the protein most significantly enriched in our model CAFs was thrombospondin‐1 (Figure 6B), which plays dual roles in modulating cell‐matrix interaction and angiogenesis with a second angiogenic regulator, PXDN (Vascular peroxidase I), also significantly enriched in our CAF‐derived sEVs. The functional roles of these proteins in angiogenesis are tissue‐dependent, but strikingly, both have recently been reported in genome‐wide screens to identify genes that significantly correlate with melanoma metastasis (Su et al. 2020; Motwani et al. 2021; Paumann‐Page et al. 2021; Smith‐Díaz et al. 2025). Other proteins identified in our TMT‐MS dataset that are differentially abundant in CAF‐derived sEV include the proteoglycans decorin and biglycan, which are depleted and enriched, respectively. Strikingly, there is a growing body of evidence from mouse studies and melanoma patient data that increased biglycan levels promote melanoma metastasis (Andrlová et al. 2017), drives tumour angiogenesis (Yamamoto et al. 2012; Maishi et al. 2016) and correlate with poor patient outcomes (Andrlová et al. 2017; Maishi et al. 2016). In contrast, decorin is reported to function in a tumour‐suppressive manner, and low levels in both melanoma tissue and circulating blood plasma have been correlated with disease progression and decreased survival rates in melanoma patients (Su et al. 2020; Appunni et al. 2025).

To further interrogate the role of thrombospondin‐1 (THBS1), which was the most significantly enriched protein in our CAF‐derived sEVs by proteomics, we performed siRNA‐mediated knockdown in our CAF model. ELISA quantification confirmed a significant increase in EV‐associated THBS1 following TGFβ1 education of fibroblasts, consistent with our proteomics dataset, and a significant reduction in THBS1 cargo in EVs from CAFs transfected with THBS1 siRNA compared with scramble control. Functionally, EVs from THBS1‐depleted CAFs induced both greater endothelial tube length in angiogenesis assays and enhanced migration in scratch assays, suggesting that THBS1 normally acts as a brake on endothelial sprouting and motility. These findings align with the reported dual and context‐dependent roles of THBS1 in vascular biology, where it can inhibit endothelial migration through CD36/CD47 signalling yet also activate latent TGFβ to promote barrier destabilisation. Our preliminary data are consistent with this dichotomy, indicating that THBS1‐depleted EVs may reduce endothelial barrier breakdown, although further experiments are required to confirm this effect. Collectively, these observations highlight THBS1 as an example of CAF EV cargo that can differentially regulate angiogenic versus barrier phenotypes, underscoring the complexity of EV‐mediated remodelling in the tumour microenvironment and pre‐metastatic niche. Analysis of our small RNA‐seq data revealed several RNAs that were present at significantly different levels in CAF‐derived sEVs compared with NDF controls (Figure 6C,D). To explore if circulating levels of these genes were altered in melanoma patients, we turned to a recent study that published small RNA‐sequencing data for 64 plasma biopsy samples from individuals with invasive melanoma or related control (Love et al. 2024). Interrogation of these data for the 7 miRNAs substantively different in our CAF‐derived sEV revealed that 5/7 miRNAs exhibited significant differences in abundance in circulating plasma. The direction of expression for these miRNAs was not consistent with changes observed in our CAF‐derived sEVs but does hint at a possible panel of miRNAs that might warrant further investigation in terms of their diagnostic power. A similar scenario emerged for the non‐miRNA genes identified in our small RNA‐sequencing data. Here, identified genes were not present in the study above, and so we leveraged our LMC cohort, which is comprised of primary tumours (35% Stage I, 50% Stage II and 15% Stage III) rather than blood plasma and cause of death, and subsequently analysed survival was exclusive to melanoma‐associated deaths (Muralidhar et al. 2019). Most genes in Figure 6E were not significant in terms of Hazard Ratios (HR); however, high expression of ZC3HAV1 and MIR148A were inversely and positively correlated with patient survival, respectively. While it is possible to extrapolate GOs from miRNA lists, these data are somewhat problematic, as they are generally derived from predicted targets, do not consider how hierarchical interactions might impact RNA targets and subsequently their functions and often miss cell‐type‐specific effects. Considering those miRNAs significantly increased in CAF‐derived sEVs, it is intriguing to note that miR‐223‐3p, miR‐148a‐3p and miR34c‐5p have been reported to be enriched in sEVs derived from a range of different cancers (Qin et al. 2021; Zhang et al. 2022; Yoshikawa et al. 2018) and are predominantly reported to function as tumour suppressors (Wan et al. 2018; Yu et al. 2019; Du et al. 2021; Fu et al. 2020; Paczkowska et al. 2020; Chen et al. 2021; Yang et al. 2023; Wei et al. 2024). We were somewhat surprised that miRNA increases in CAF‐derived sEVs were reported to be tumour‐suppressive; however, an emerging concept regarding the release of sEVs within the TME is that cancer cells and CAFs may exploit the packaging of miRNAs into sEVs and their subsequent release to deplete miRNAs that negatively regulate cell growth, thereby promoting a cancer‐associated phenotype (Kanlikilicer et al. 2016; van der Merwe et al. 2025). This mechanism may also help reconcile our observation that two RNAs enriched in CAF‐derived EVs, ZC3HAV1 and MIR148A, showed opposite associations with patient survival in Kaplan–Meier analysis, suggesting that selective EV export could differentially deplete protective or deleterious transcripts from the CAF cell. Evidence to support this model is on the increase, for example, a recent study showed that non‐small cell lung cancer cells escape the suppressive effects of the tumour‐suppressive miRNA, miR‐4732‐3p, by selectively packaging them into sEVs, thereby supporting tumour progression (Zhuang et al. 2024). How cells selectively export miRNA is still not fully understood, but RNA‐binding proteins are likely culprits, with hnRNPK implicated in the previous example and YBX1 recently reported to selectively package miR‐233 into sEVs (Liu et al. 2021).

In summary, more work is needed to determine the contribution of CAF‐derived sEV to circulating levels of key modulators of the ECM and tumour vascular network. With the development of emerging pipelines for deconvolution of tissue‐ and cell‐type‐specific extracellular vesicle abundances (Gupta et al. 2025; Larsen et al. 2024), we are approaching a point where we can identify which tumour cells contribute significant levels of pro‐tumourigenic biomolecules to the circulation. Our data suggest that CAF‐derived sEVs may play a significant role in this process, positioning them as an attractive therapeutic target. Through omics analysis of model CAF‐derived EVs, combined with phenotypic data on their impact on brain endothelial biology, our data suggest some compelling insights into melanoma brain tropism. However, we recognise several limitations of this study, including reliance on a simplified CAF model, use of monocultures rather than integrated BBB systems, lack of extended CAF marker and protein‐level EndMT validation, and reliance on pharmacological EV inhibition (GW4869) rather than genetic approaches such as Rab27 knockdown. Moreover, it will be crucial to explore how these findings translate to a clinical setting, and we are currently performing longitudinal plasma sampling in melanoma patients to assess whether CAF‐specific miRNAs or proteins can serve as predictors of metastatic progression.

## Author Contributions

**M. Shelton**: conceptualization, data curation, formal analysis, investigation, methodology, validation, visualization, writing – original draft, writing – review and editing. **C. A. Anene**: conceptualization, data curation, formal analysis, investigation, methodology, software, validation, visualization, writing – original draft, writing – review and editing. **J. Nsengimana**: data curation, formal analysis, validation, visualization, writing – review and editing. **M. K. Eldahshoury**: conceptualization, formal analysis, investigation, methodology, validation, visualization, writing – review and editing. **J. G. Gillet‐Woodley**: formal analysis, investigation, validation, writing – review and editing. **B. Keane**: formal analysis, validation, visualization, writing – review and editing. **W. Roberts**: conceptualization, funding acquisition, supervision. **J. Newton‐Bishop**: conceptualization, funding acquisition, supervision, writing – original draft, writing – review and editing. **J. R. Boyne**: conceptualization, data curation, formal analysis, funding acquisition, methodology, project administration, resources, supervision, validation, visualization, writing – original draft, writing – review and editing.

## Conflicts of Interest

The authors declare no conflicts of interest.

## Supporting information



Supplementary Information

Supplementary Information

## Data Availability

The data that supports the findings of this study are available in the supplementary material of this article
