# Supplementary material for: Extracellular Vesicles From a Model of Melanoma Cancer‐Associated Fibroblasts Induce Changes in Brain Microvascular Cells Consistent With Pre‐Metastatic Niche Priming
Source: J Extracell Biol. 2025 Oct 30;4(11):e70094. doi: 10.1002/jex2.70094 (PMC12575059; doi:10.1002/jex2.70094)
Supplement: Supplementary file 2 — Supplementary Information [file JEX2-4-e70094-s001.pdf]

Fig. S1

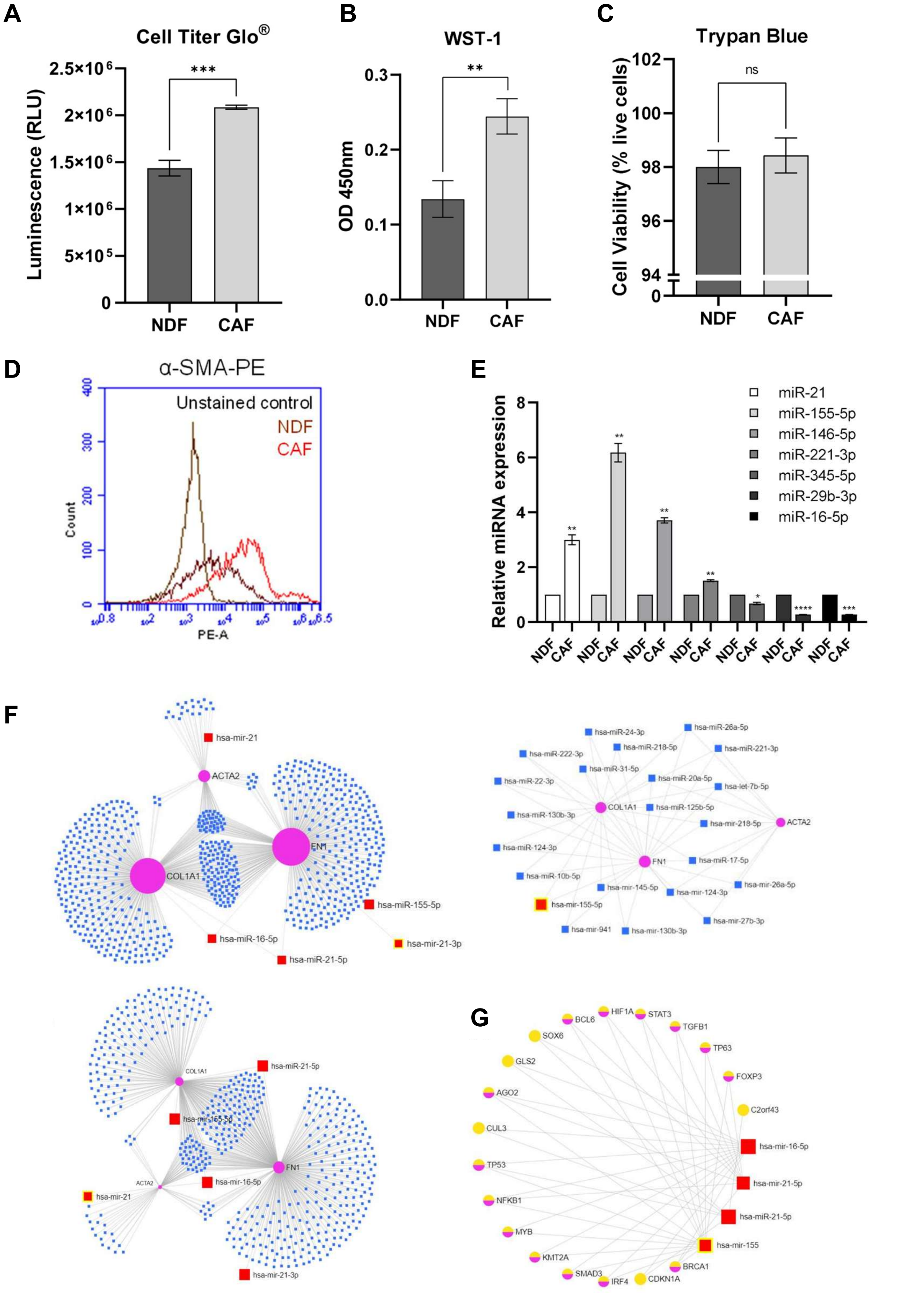
A
Cell Titer Glo®
Luminescence (RLU)
NDF
CAF
B
WST-1
OD 450nm
NDF
CAF
C
Trypan Blue
Cell Viability (% live cells)
ns
NDF
CAF
D
α-SMA-PE
Unstained control
NDF
CAF
Count
PE-A
E
Relative miRNA expression
miR-21
miR-155-5p
miR-146-5p
miR-221-3p
miR-345-5p
miR-29b-3p
miR-16-5p
F
hsa-mir-21
ACTA2
FN1
COL1A1
hsa-miR-155-5p
hsa-miR-16-5p
hsa-mir-21-3p
hsa-miR-21-5p
G
hsa-mir-16-5p
hsa-mir-21-5p
hsa-miR-21-5p
hsa-mir-155

H

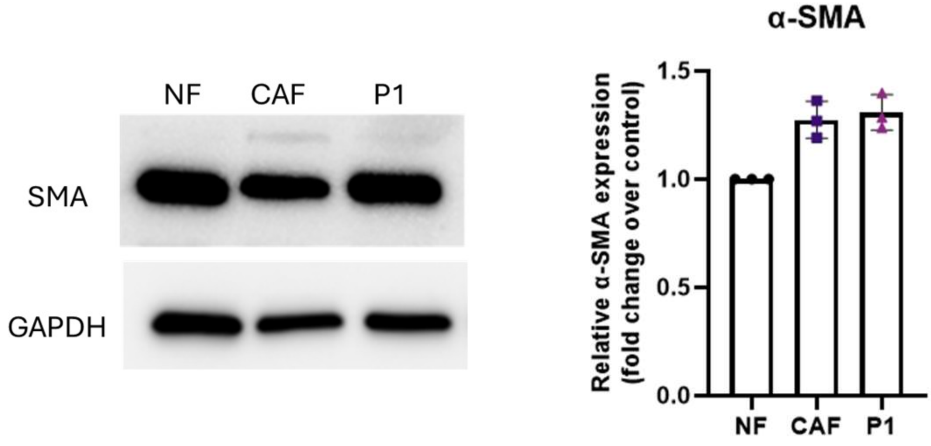
NF
CAF
P1
SMA
GAPDH
α-SMA
Relative α-SMA expression
(fold change over control)
1.5
1.0
0.5
0.0
NF
CAF
P1

Fig. S2

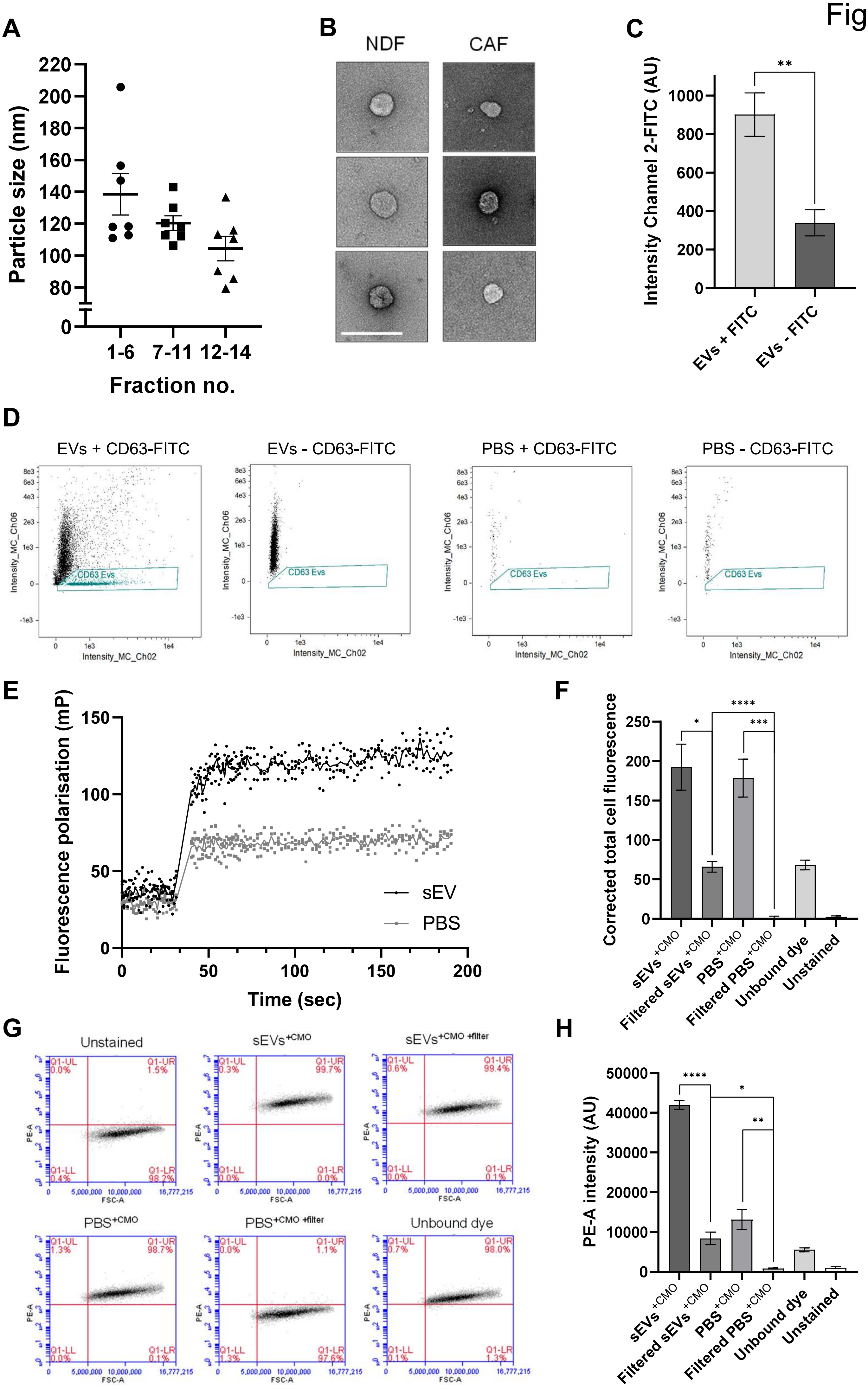
A
Particle size (nm)
Fraction no.
1-6
7-11
12-14
B
NDF
CAF
C
Intensity Channel 2-FITC (AU)
**
EVs + FITC
EVs - FITC
D
EVs + CD63-FITC
EVs - CD63-FITC
PBS + CD63-FITC
PBS - CD63-FITC
CD63 Evs
Intensity_MC_Ch06
Intensity_MC_Ch02
E
Fluorescence polarisation (mP)
Time (sec)
sEV
PBS
F
Corrected total cell fluorescence
sEVs +CMO
Filtered sEVs +CMO
PBS +CMO
Filtered PBS +CMO
Unbound dye
Unstained
G
Unstained
sEVs+CMO
sEVs+CMO +filter
PBS+CMO
PBS+CMO +filter
Unbound dye
PE-A
FSC-A
H
PE-A intensity (AU)

Fig. S2 cont.

I

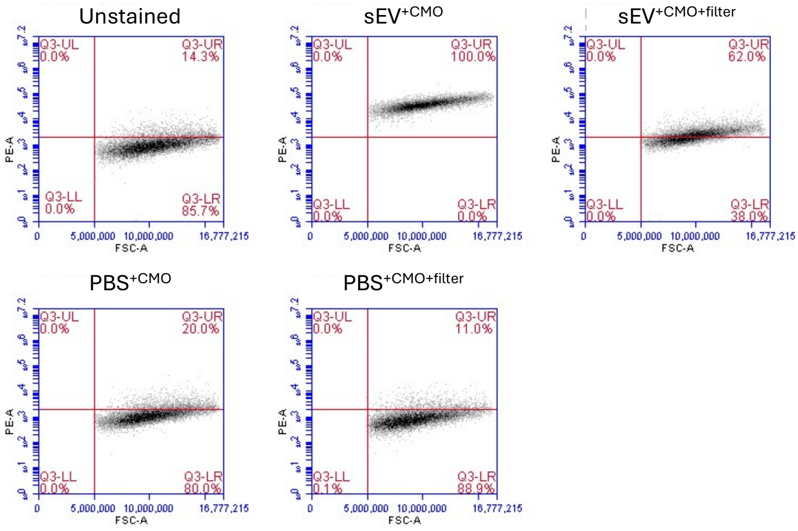

J

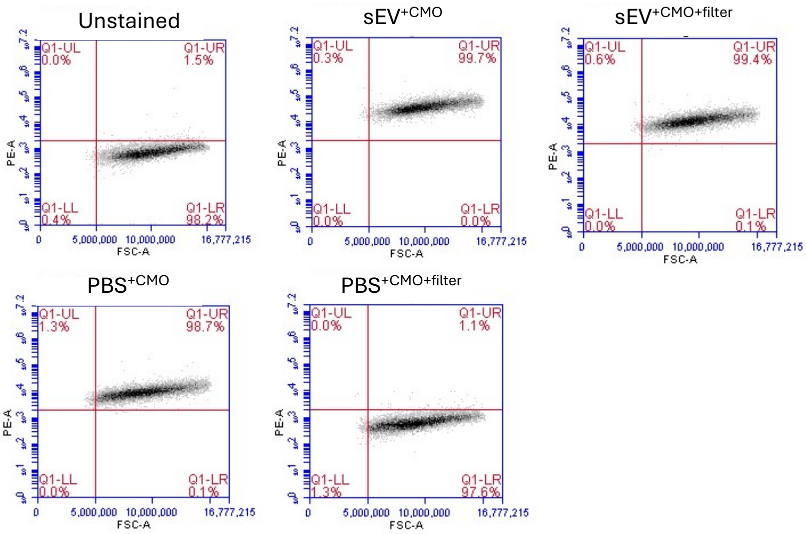

K

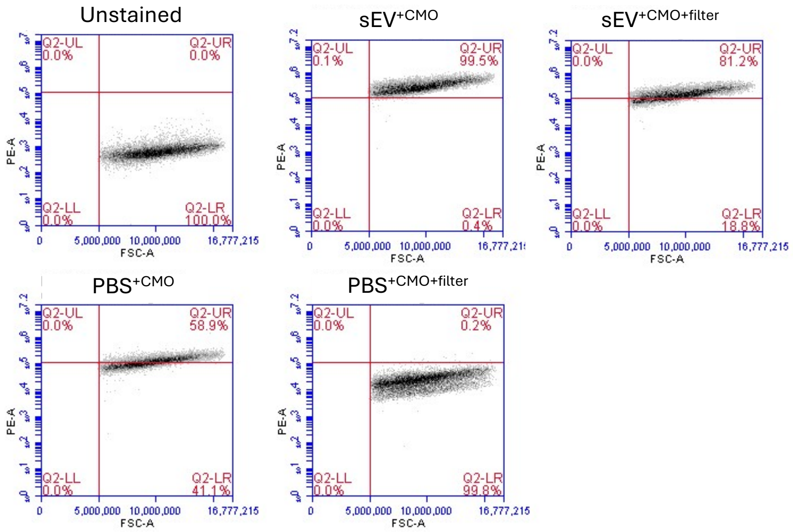

L

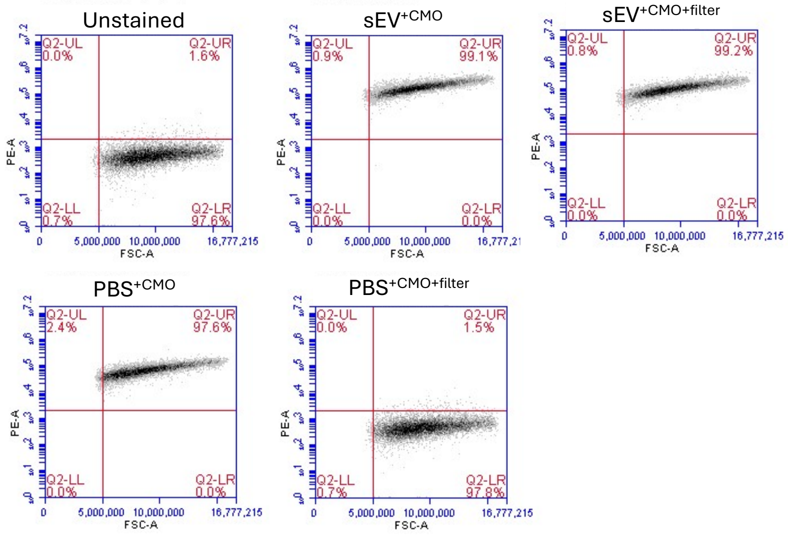

Fig. S3

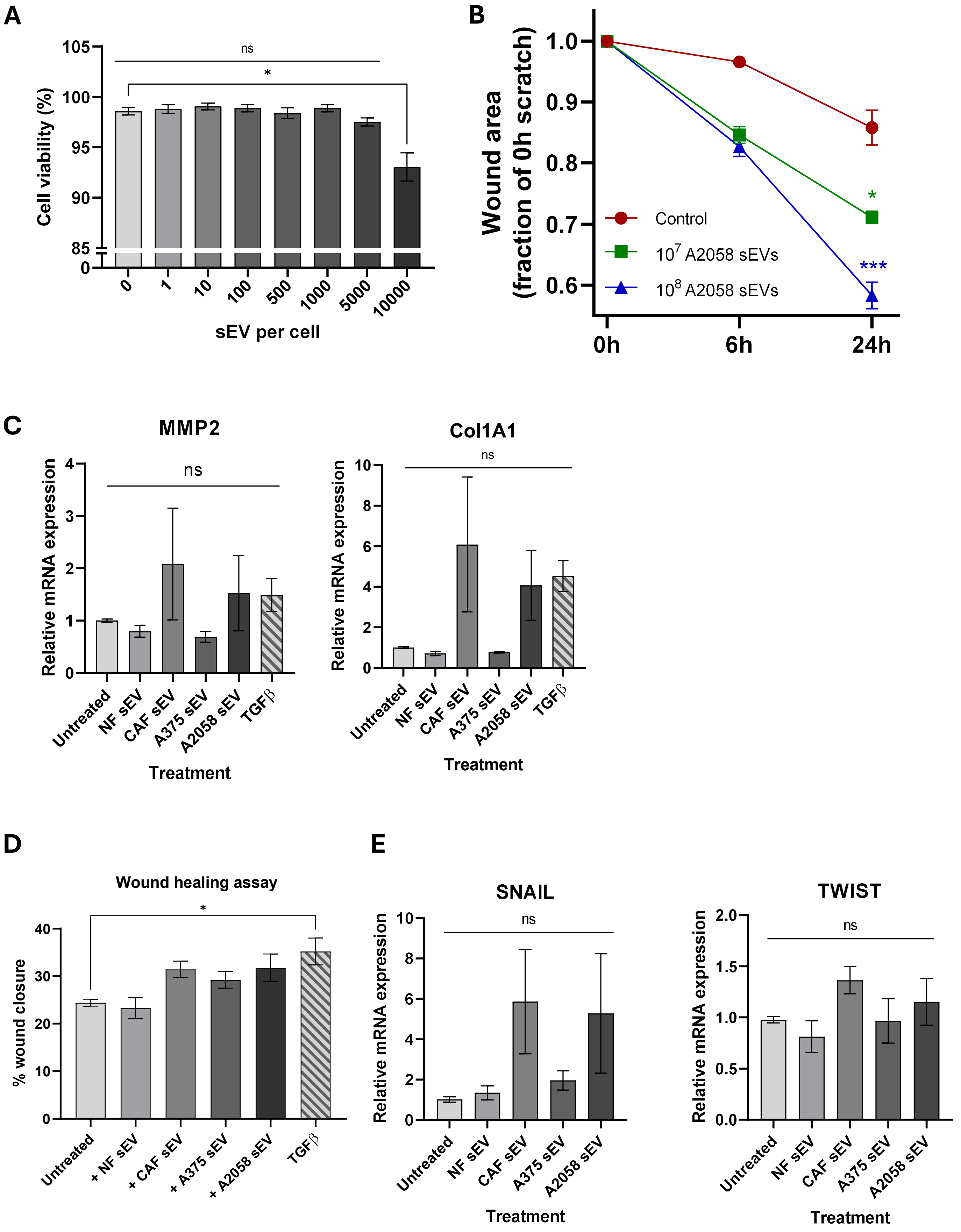

A
Cell viability (%)
ns
*
sEV per cell
0
1
10
100
500
1000
5000
10000
B
Wound area
(fraction of 0h scratch)
Control
10^7 A2058 sEVs
10^8 A2058 sEVs
*
***
0h
6h
24h
C
MMP2
Col1A1
Relative mRNA expression
ns
Untreated
NF sEV
CAF sEV
A375 sEV
A2058 sEV
TGFβ
Treatment
D
Wound healing assay
% wound closure
*
Untreated
+ NF sEV
+ CAF sEV
+ A375 sEV
+ A2058 sEV
TGFβ
E
SNAIL
TWIST
Relative mRNA expression
ns
Untreated
NF sEV
CAF sEV
A375 sEV
A2058 sEV
Treatment

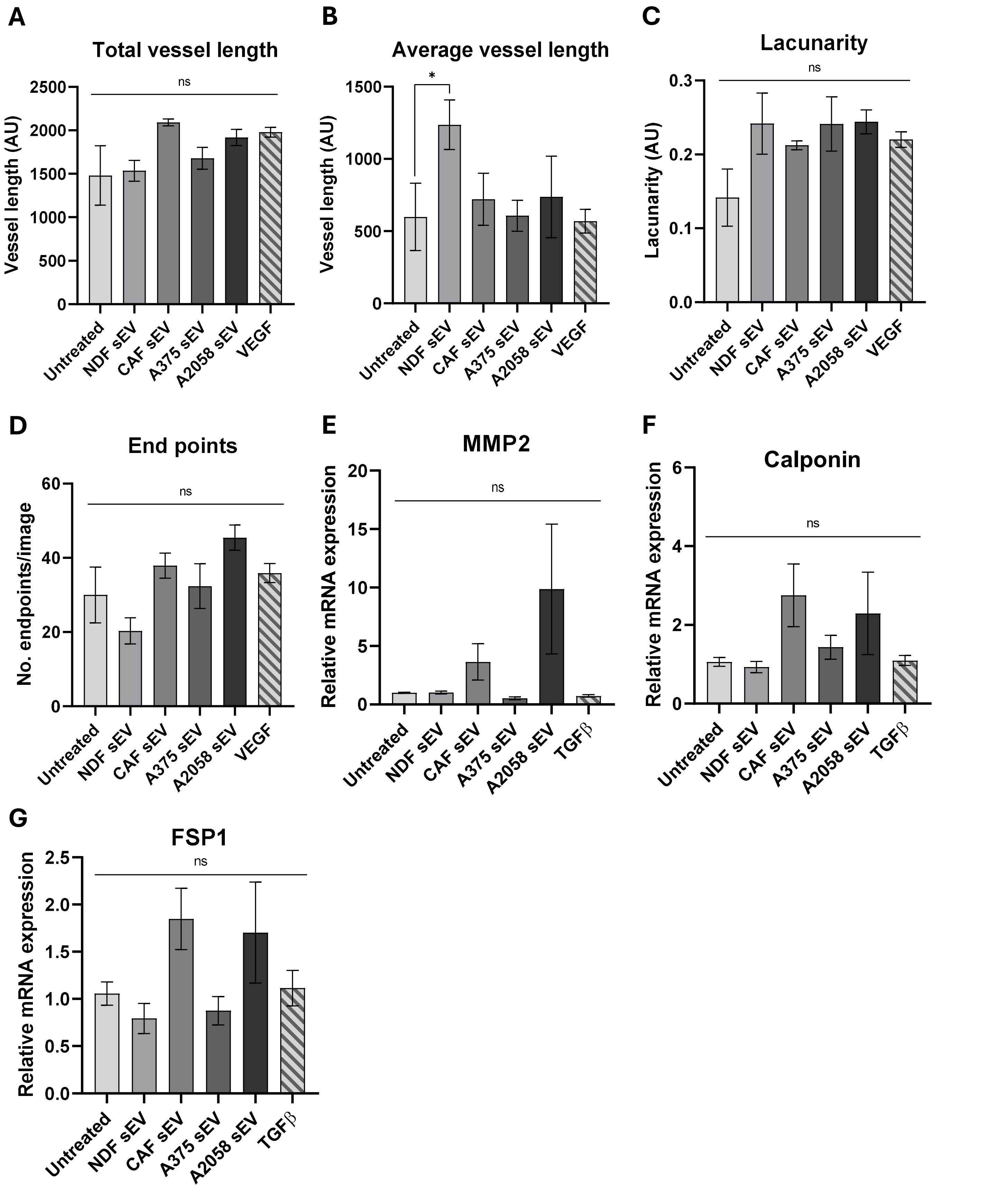
A
Total vessel length
Vessel length (AU)
ns
B
Average vessel length
Vessel length (AU)
*
C
Lacunarity
Lacunarity (AU)
ns
D
End points
No. endpoints/image
ns
E
MMP2
Relative mRNA expression
ns
F
Calponin
Relative mRNA expression
ns
G
FSP1
Relative mRNA expression
ns
Untreated
NDF sEV
CAF sEV
A375 sEV
A2058 sEV
VEGF
TGFβ

Fig. S5

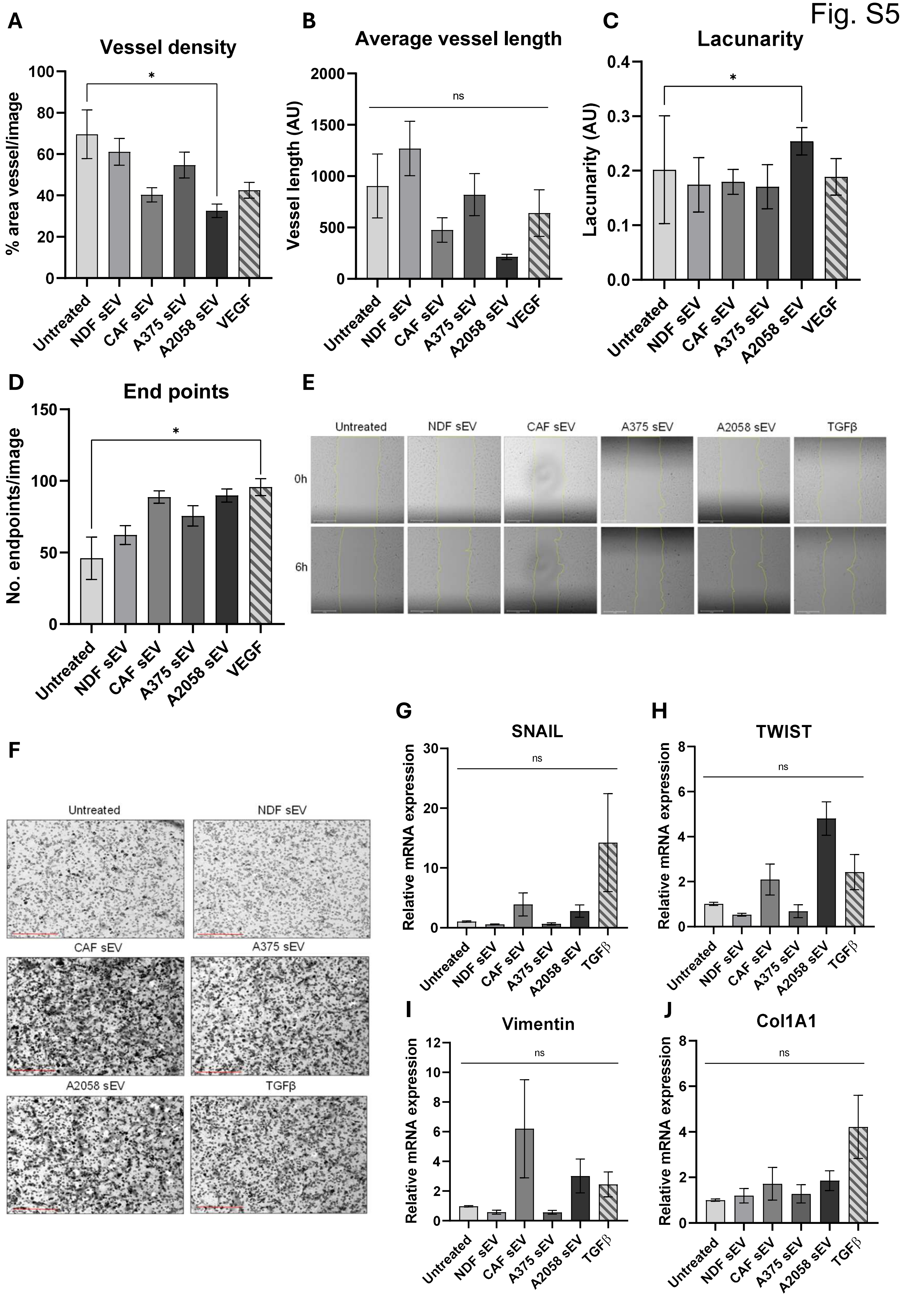
A
Vessel density
% area vessel/image
Untreated
NDF sEV
CAF sEV
A375 sEV
A2058 sEV
VEGF
B
Average vessel length
Vessel length (AU)
ns
C
Lacunarity
Lacunarity (AU)
D
End points
No. endpoints/image
E
Untreated
NDF sEV
CAF sEV
A375 sEV
A2058 sEV
TGFβ
0h
6h
F
G
SNAIL
Relative mRNA expression
H
TWIST
I
Vimentin
J
Col1A1

Fig. S5 cont.

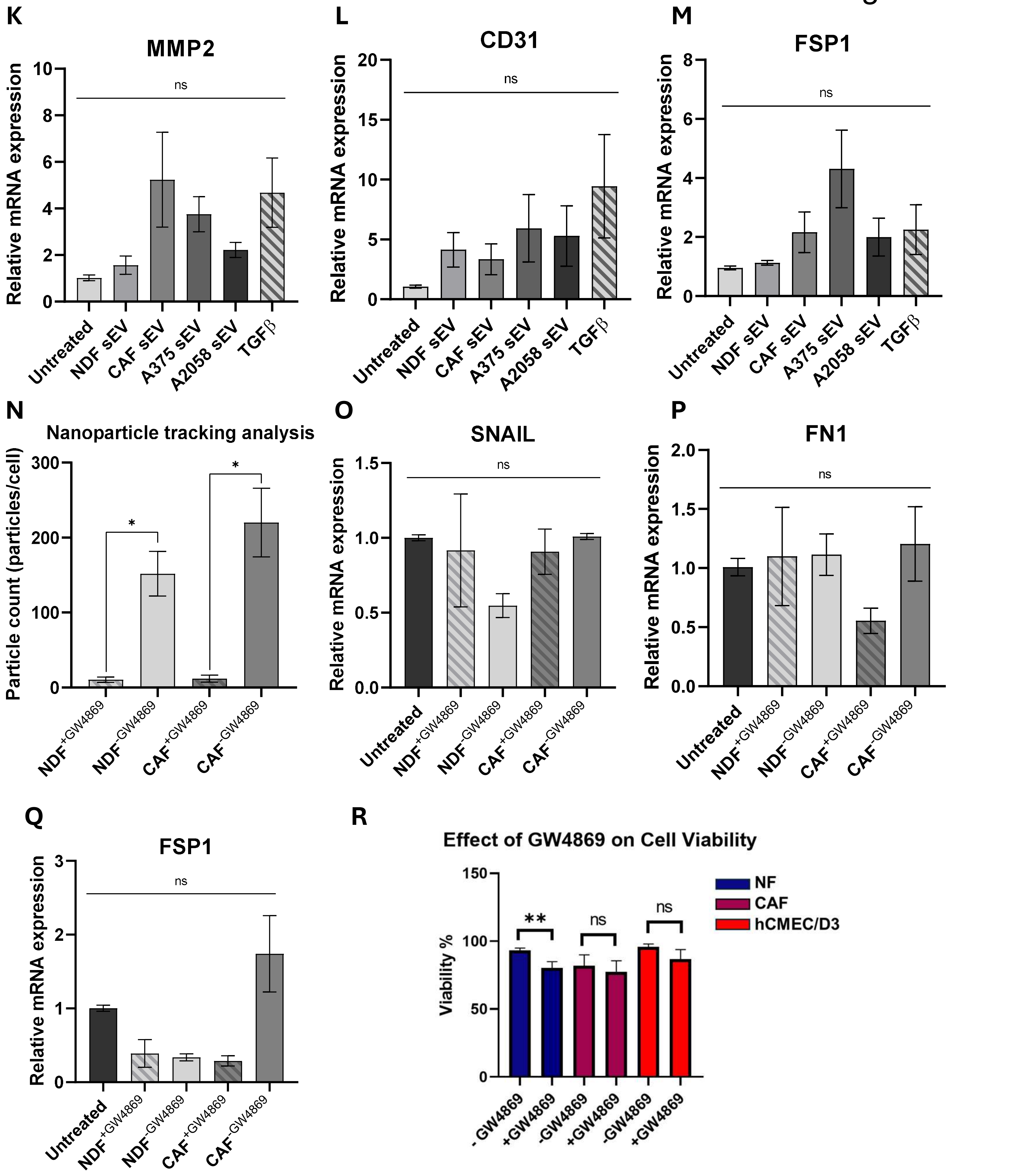
K
MMP2
Relative mRNA expression
ns
Untreated
NDF sEV
CAF sEV
A375 sEV
A2058 sEV
TGFβ
L
CD31
M
FSP1
N
Nanoparticle tracking analysis
Particle count (particles/cell)
NDF+GW4869
NDF-GW4869
CAF+GW4869
CAF-GW4869
O
SNAIL
P
FN1
Q
FSP1
R
Effect of GW4869 on Cell Viability
Viability %
NF
CAF
hCMEC/D3
- GW4869
+GW4869
-GW4869
+GW4869
-GW4869
+GW4869

Fig. S6

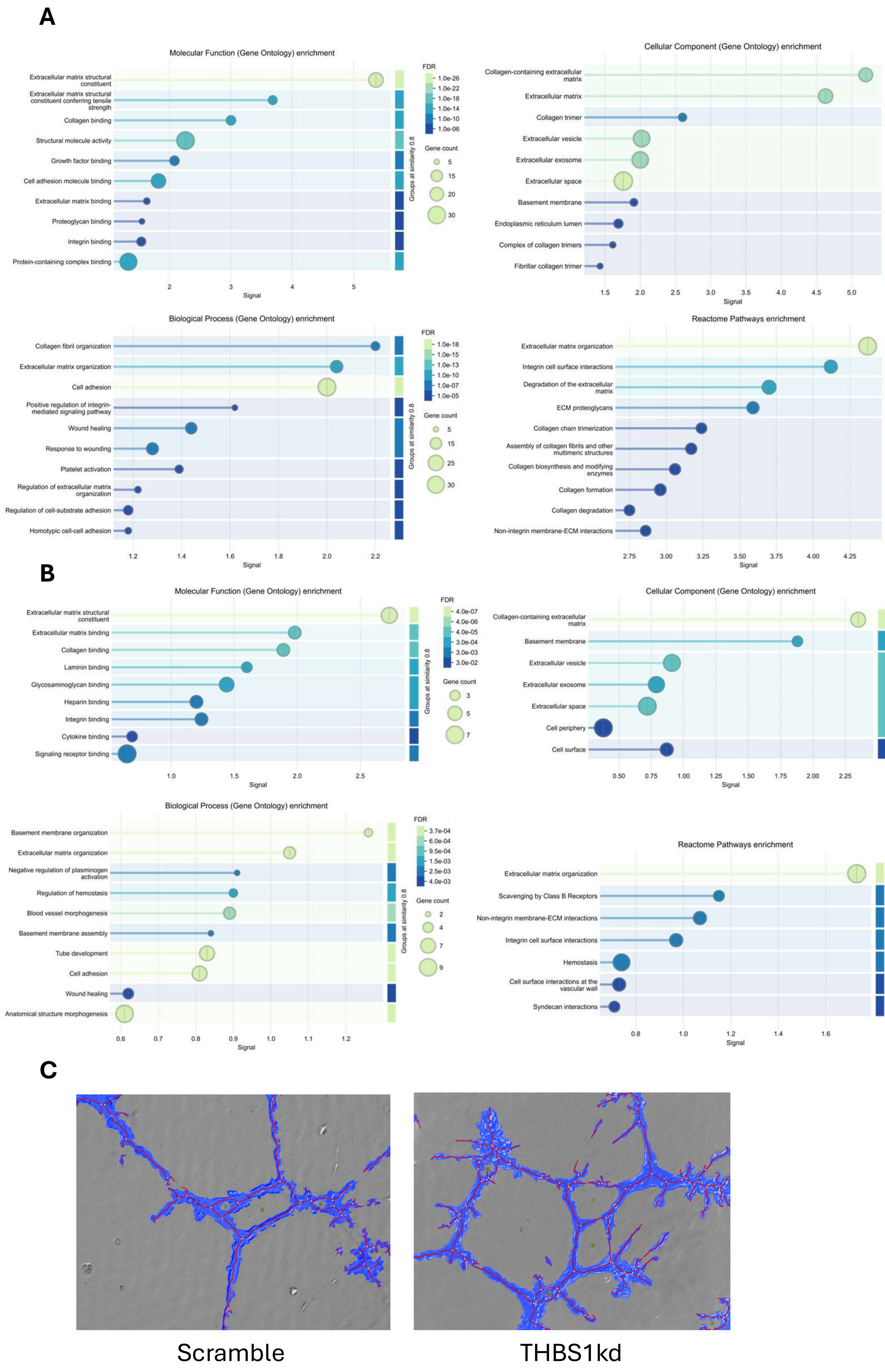
A
Molecular Function (Gene Ontology) enrichment
Extracellular matrix structural constituent
Extracellular matrix structural constituent conferring tensile strength
Collagen binding
Structural molecule activity
Growth factor binding
Cell adhesion molecule binding
Extracellular matrix binding
Proteoglycan binding
Integrin binding
Protein-containing complex binding
Signal
FDR
Gene count
Groups at similarity 0.8
Cellular Component (Gene Ontology) enrichment
Collagen-containing extracellular matrix
Extracellular matrix
Collagen trimer
Extracellular vesicle
Extracellular exosome
Extracellular space
Basement membrane
Endoplasmic reticulum lumen
Complex of collagen trimers
Fibrillar collagen trimer
Biological Process (Gene Ontology) enrichment
Collagen fibril organization
Extracellular matrix organization
Cell adhesion
Positive regulation of integrin-mediated signaling pathway
Wound healing
Response to wounding
Platelet activation
Regulation of extracellular matrix organization
Regulation of cell-substrate adhesion
Homotypic cell-cell adhesion
Reactome Pathways enrichment
Extracellular matrix organization
Integrin cell surface interactions
Degradation of the extracellular matrix
ECM proteoglycans
Collagen chain trimerization
Assembly of collagen fibrils and other multimeric structures
Collagen biosynthesis and modifying enzymes
Collagen formation
Collagen degradation
Non-integrin membrane-ECM interactions
B
Molecular Function (Gene Ontology) enrichment
Extracellular matrix structural constituent
Extracellular matrix binding
Collagen binding
Laminin binding
Glycosaminoglycan binding
Heparin binding
Integrin binding
Cytokine binding
Signaling receptor binding
Cellular Component (Gene Ontology) enrichment
Collagen-containing extracellular matrix
Basement membrane
Extracellular vesicle
Extracellular exosome
Extracellular space
Cell periphery
Cell surface
Biological Process (Gene Ontology) enrichment
Basement membrane organization
Extracellular matrix organization
Negative regulation of plasminogen activation
Regulation of hemostasis
Blood vessel morphogenesis
Basement membrane assembly
Tube development
Cell adhesion
Wound healing
Anatomical structure morphogenesis
Reactome Pathways enrichment
Extracellular matrix organization
Scavenging by Class B Receptors
Non-integrin membrane-ECM interactions
Integrin cell surface interactions
Hemostasis
Cell surface interactions at the vascular wall
Syndecan interactions
C
Scramble
THBS1kd

Fig. S6 cont.

D

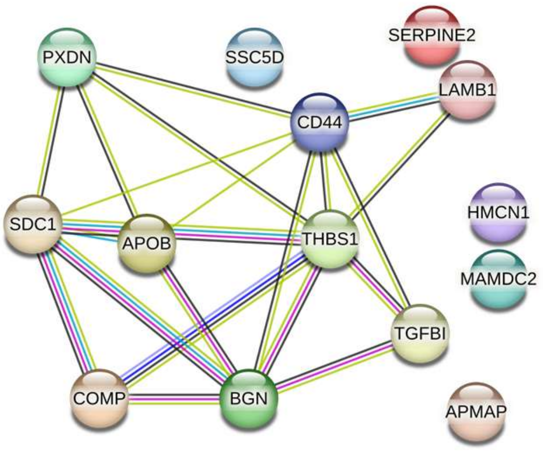

E

Gene (probe)	HR (95% CI)	Cox model Pvalue
**MIR148A (ilmn_3308723)**	**0.75 (0.57, 1.0)**	**0.048**
**ZC3HAV1 (ilmn_1724837)**	**1.58 (1.19, 2.10)**	**0.0016**
MIR223 (ilmn_3308936)	0.94 (0.71, 1.24)	0.66
MIR302C (ilmn_3308265)	0.80 (0.61, 1.10)	0.13
MIR326 (ilmn_3310980)	1.05 (0.80, 1.39)	0.72
MR1 (ilmn_2167416)	1.20 (0.91, 1.60)	0.21
SNORD83A (ilmn_1653927)	0.85 (0.64, 1.13)	0.26
VTRNA1-1 (ilmn_3309759)	1.15 (0.87, 1.52)	0.34
